# Mechanism-Dependent Selectivity: Fluorocyclization of Unsaturated Carboxylic Acids or Alcohols by Hypervalent Iodine

**DOI:** 10.3389/fchem.2022.897828

**Published:** 2022-05-10

**Authors:** Jiaqi Su, Siwei Shu, Yinwu Li, Yong Chen, Jinxiang Dong, Yan Liu, Yanxiong Fang, Zhuofeng Ke

**Affiliations:** ^1^ School of Chemical Engineering and Light Industry, Guangdong University of Technology, Guangzhou, China; ^2^ PCFM Lab, School of Materials Science and Engineering, Sun Yat-sen University, Guangzhou, China; ^3^ Guangdong Provincial Key Laboratory of Chemical Measurement and Emergency Test Technology, Institute of Analysis, Guangdong Academy of Sciences (China National Analytical Center, Guangzhou), Guangzhou, China; ^4^ Guangdong Provincial Key Laboratory of Plant Resources Biorefinery, Guangzhou, China; ^5^ Guangdong Provincial Key Laboratory of Optical Chemicals, XinHuaYue Group, Maoming, China

**Keywords:** fluorination, cyclization, 6-endo, 5-oxo, hypervalent iodine, mechanism, DFT

## Abstract

To understand the unprecedented difference between *6-endo* and *5-exo* selectivity in hypervalent iodine (III) promoted fluorocyclization of unsaturated carboxylic acids or alcohols by difluoroiodotoluene, density functional theory (DFT) studies have been performed to systematically compare both the previous proposed “fluorination first and cyclization later” mechanism and the alternative “cyclization first and fluorination later” mechanism. Our results revealed that the selectivity is mechanism-dependent. The unsaturated alcohol prefers the fluorination first and the *6-endo-tet* cyclization later pathway, leading to the experimentally observed *6-endo* ether product. In contrast, the unsaturated carboxylic acid plausibly undergoes the *5-exo-trig* cyclization first and the fluorination later to the experimentally observed *5-exo* lactone product. The p*K*_a_ property of the functional group of the substrate is found to play a key role in determining the reaction mechanism. The provided insights into the mechanism-dependent selectivity should help advance the development of fluorocyclization reactions with hypervalent iodine reagents.

## Introduction

Owing to their unique physical and chemical properties, organofluorine compounds are widely applied in pharmaceuticals (Ma and Cahard, 2008), agrochemicals (Theodoridis, 2006), functional materials (Preshlock et al., 2016), and many other areas (O’Hagan and Deng, 2015; Yang et al., 2015; Zhou Y. et al., 2016). For example, approximately 30% of all agrochemicals and 20% of all pharmaceuticals contain fluorine atom(s) in their structures (Berger et al., 2011). Incorporating fluorine atom(s) into new compounds can improve molecular efficiency, permeability, lipophilicity, and biological activity (Zhu et al., 2018). However, there are few organofluorine compounds known in nature; therefore, developing new methods of synthesizing new organofluorine compounds and understanding their reaction mechanism would be critically important (Yoneda, 2004; Zhou et al., 2016a; Kohlhepp and Gulder, 2016; Fustero et al., 2018; Sreenithya and Sunoj, 2019; Han and Zhang, 2020; Saito, 2020; Wang et al., 2020; Zheng and Xue, 2020; Jalil et al., 2021).

**SCHEME 1:**
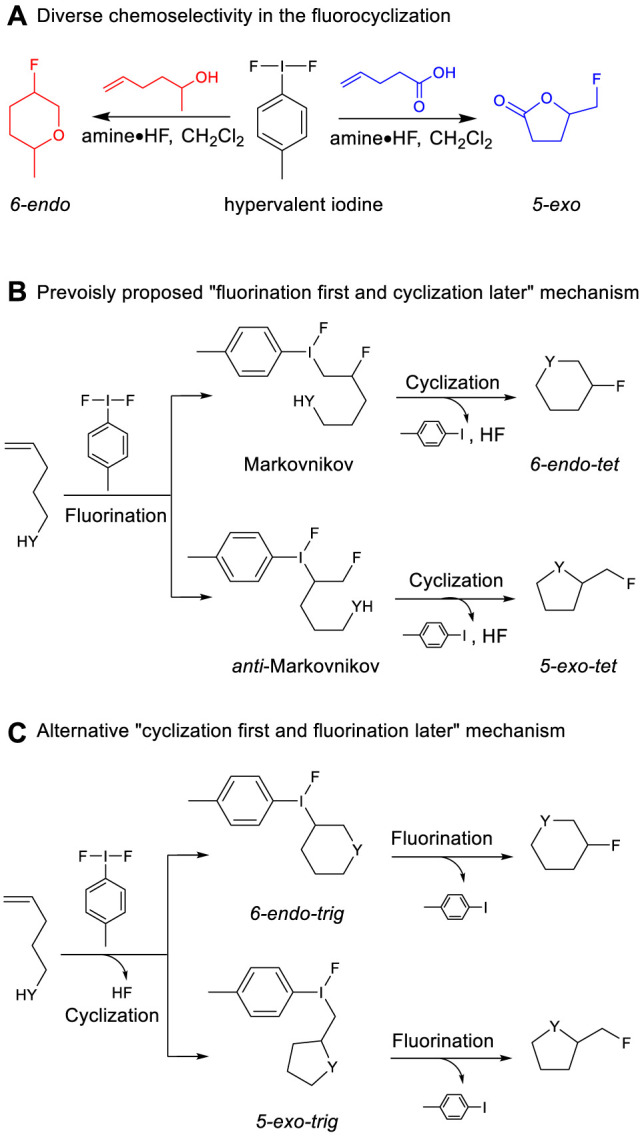
Fluorocyclization of unsaturated carboxylic acids or alcohols by hypervalent iodine.

In recently developed methods of synthesizing organofluorine compounds, hypervalent iodine reagents gained increasing attention (Yoshimura and Zhdankin, 2016). For example, Charpentier et al. (2015) have applied Togni reagents to introduce trifluoromethyl into alkenyls. Ilchenko et al. (2016) reported introducing fluorine atom(s) into alkenyls using fluoro-iodoxole (Mai et al., 2018). Sawaguchi et al. (2000) utilized difluoroiodotoluene to introduce fluorine atom(s) into unsaturated alcohols and unsaturated carboxylic acids, as shown in Scheme 1A. This interesting transformation resulted in unprecedented different selectivity (*6-endo vs. 5-exo*, Scheme 1A) between unsaturated alcohol and unsaturated carboxylic acid substrates. It was proposed that the hypervalent iodine reagent promotes the fluorination of the C=C double bond, and then the nucleophilic substitution furnishes the cyclization products in *6-endo* or *5-exo* selectivity, as shown in Scheme 1B (the “fluorination first and cyclization later” mechanism). It is desired to understand what is the origin behind this unprecedented diverse selectivity. Although it was suggested that the *6-endo* product could be formed from the *5-exo* intermediate *via* a cyclo-oxonium species, this kind of highly constrained oxonium may be suspected to be thermodynamically plausible. Interestingly, an alternative mechanism was also proposed, where the intramolecular cyclization occurs first by the nucleophilic attack of the functional group to the activated alkene, followed by fluorination later, as shown in Scheme 1C (the “cyclization first and fluorination later” mechanism) (Cui et al., 2014; Yang et al., 2015; Han et al., 2017; Kitamura et al., 2017; Farshadfar et al., 2020). In other similar reactions, the *6-endo vs. 5-exo* phenomenon also attracted many interests (Liu et al., 2014; Kong et al., 2015; Zhao et al., 2017; Zhao et al., 2018; Riley et al., 2021). However, the origin of this selectivity is still ambiguous. Further insights are desired to clarify the understanding of the reaction mechanism, which should be system-dependent on not only the substrates but also the hypervalent iodine reagent itself or even the reaction conditions. Specifically, in the works by Sawaguchi et al. (2000) and Yoneda (2004), the same difluoro-iodoarenes reagents promoted diverse selective reactions in similar conditions, which should be an ideal model to study the difference in selectivity and understand its mechanistic origin. In our previous study (Shu et al., 2019), we have uncovered the mechanism that the hypervalent iodine reagent promoted fluorocyclization of unsaturated alcohols *via* double acid activation. Herein, we utilized DFT studies to clarify the mechanism-dependent selectivity of Hara and Yoneda’s system. We also investigated the role of the acid dissociation constant (p*K*_a_) of the functional group on the reaction mechanism.

## Calculation Details

All calculations were carried out with the Gaussian 09 program (Frisch et al., 2013). Geometry optimizations and frequency calculations were carried out with B97D functional (Grimme, 2006; Lu et al., 2012), LANL2DZ containing the corresponding basis set augmented with d polarization and p diffuse functions for I atom (Couty and Hall, 1996), and the 6-31G (d, p) for the other atoms. Higher Level single-point energy calculations were carried out in the solvent of dichloromethane with the SMD model (Marenich et al., 2009), M06-2X functional was employed (Zhao and Truhlar, 2008; Walker et al., 2013), SDD basis (Andrae et al., 1990) for iodine atom, and 6-311++G (d, p) for other atoms. The NBO analysis was carried out with M06-2X functional, the SDD model for iodine, and 6-311++G (d,p) for other atoms; the solvent is dichloromethane and with the SMD calculated model. Considering the entropic contribution is overestimated due to the ignorance of the suppression effect of solvent on the translational and rotational freedoms of the reactants, the MHP scheme proposed by Martin et al. (1998) is adopted. All the pictures of structures were generated by CYLview (Legault, 2009).

## Results and Discussion

**FIGURE 1:**
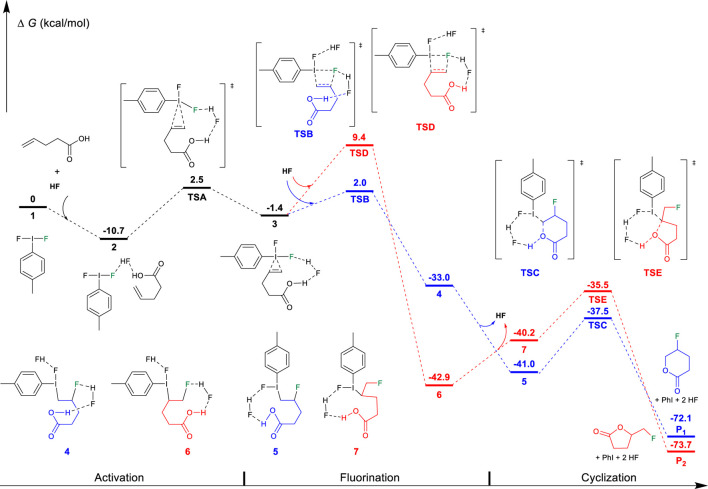
Free-energy profiles for the fluorocyclization of unsaturated carboxylic acid *via* the “fluorination first and cyclization later” mechanism. The preferred pathway is shown in blue.

**FIGURE 2:**
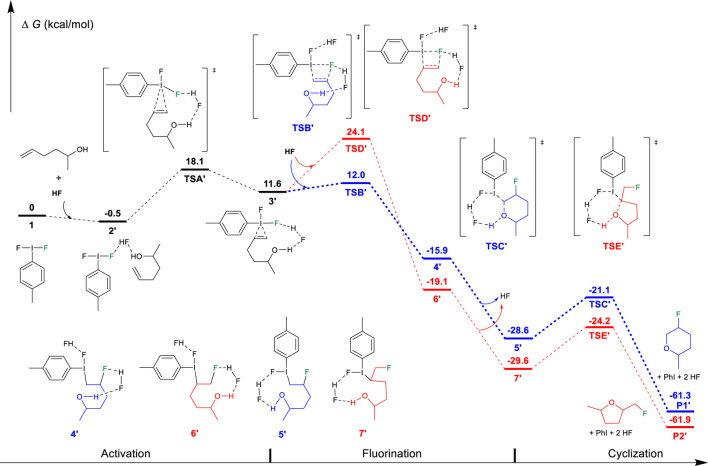
Free-energy profiles for the fluorocyclization of unsaturated alcohol *via* the “fluorination first and cyclization later” mechanism. The preferred pathway is shown in blue.

**FIGURE 3:**
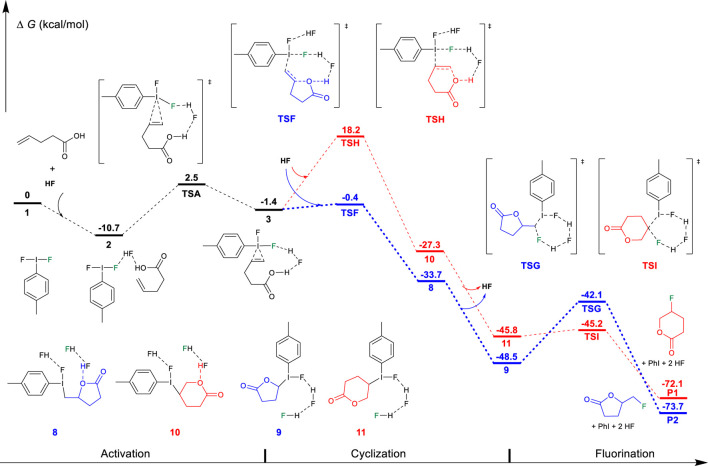
Free-energy profiles for the fluorocyclization of unsaturated carboxylic acid *via* the “cyclization first and fluorination later” mechanism. The preferred pathway is shown in blue.

**FIGURE 4:**
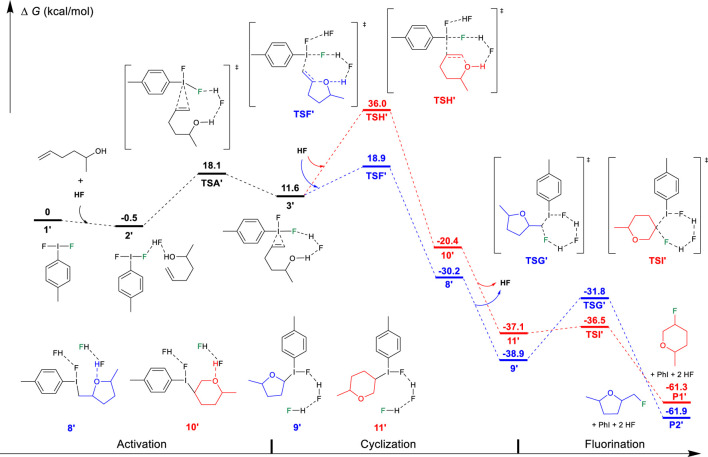
Free-energy profiles for the fluorocyclization of unsaturated alcohol *via* the “cyclization first and fluorination later” mechanism. The preferred pathway is shown in blue.

On the basis of the aforementioned mechanistic understanding, the hypervalent iodine promoted fluorocyclization generally includes three stages: the activation of alkene, fluorination, and cyclization. DFT calculations have been performed to provide insights to understand the selectivity and the origin of the reaction by comprehensively comparing the mechanistic difference in “fluorination first and cyclization later” and the “cyclization first and fluorination later” (Scheme 1). The free energy profiles for the fluorocyclization of unsaturated carboxylic acid *via* the “fluorination first and cyclization later” mechanism or the “cyclization first and fluorination later” mechanism are depicted in Figures 1, 2, respectively. The results of the fluorocyclization of unsaturated alcohol through the “fluorination first and cyclization later” mechanism or the “cyclization first and fluorination later” mechanism are shown in Figures 3, 4, respectively.

### “Fluorination First and Cyclization Later” Mechanism

According to previous studies (Shu et al., 2019), this hypervalent iodine-promoted fluorocyclization reaction preferred a metathesis fluorination mechanism *via* activated iodine (III)-π intermediate in which the Bronsted acid double-activation mode plays an important role due to the use of excess amine·HF. The formed iodine (III) iranium intermediate was predicted to be much higher in free energy (Shu et al., 2019). Therefore, in the “fluorination first and cyclization later” mechanism, the unsaturated carboxylic alcohol is first activated by the difluoroiodotoluene *via* TSA, producing an iodine (III)-π intermediate 3, as shown in Figure 1. In intermediate 3, the I-C bond lengths between iodine and alkene are calculated to be 2.699/2.895 Å, indicating a typical iodine (III)-π activation instead of an iodine (III) iranium activation (Zhou et al., 2016b; Zhang et al., 2017; Shu et al., 2019). The free energy barrier of the activation step is 13.2 kcal/mol.

After the activation, with the assistance of another HF as the Brønsted acid, the fluorination of the C=C double undergoes the metathesis mechanism *via* transition state TSB or TSD. TSB leads to the Markovnikov product 4, while TSD results in the *anti-*Markovnikov product 6. TSB (2.0 kcal/mol) is preferred over TSD (9.4 kcal/mol), which is in good agreement with Markovnikov’s rule. Both transition states have similar four-membered ring structures, where the I-C, I-F, C=C, and C-F bond lengths are calculated to be 2.514/2.575/1.400/2.342 Å in TSB and 2.611/2.536/1.397/2.227 Å in TSD, respectively. It can be found that the interaction between hypervalent iodine and alkene is stronger in TSB with longer I-F and C=C bond lengths, leading to a stronger I-C interaction (2.514 Å). However, the free energy preferred transition state TSB leads to the final *6-endo-tet* annulation product P1 *via* the transition state TSC (−37.5 kcal/mol). This is a kinetically and thermodynamically facile step (Δ*G* ^⧧^ = 3.5 kcal/mol); however, the predicted selectivity of the product is in sharp contrast to the experimental observation of a *5-exo* product for the unsaturated carboxylic acid substrates. TSE has a relatively high ring constraint as compared to TSC due to the role of lactone in the five-membered ring transition state. Out of expectation, the annulation (6→7→TSE→P2) leads to the experimentally observed *5-exo* product P2, which is predicted by the DFT results to be unfavored along the “fluorination first and cyclization later” mechanism (TSD, 9.4 kcal/mol). This strongly indicates that the “fluorination first and cyclization later” mechanism cannot explain the selectivity of the unsaturated carboxylic acid in difluoroiodotoluene-promoted fluorocyclization.

On the contrary, for unsaturated alcohols, this “fluorination first and cyclization later” mechanism can explain the experimental phenomenon well. As shown in Figure 2, the activation of the unsaturated alcohol also leads to an iodine (III)-π intermediate 3′ (Zhou et al., 2016a; Zhang et al., 2017; Shu et al., 2019). The located 3′ structure has a very similar I···alkene interaction (2.685/2.871 Å) to that in structure 3. After the activation, the fluorination of the C=C bond of the unsaturated alcohol also prefers the Markovnikov transition state TSB′ (12.0 kcal/mol) in comparison to the higher *anti-*Markovnikov transition state TSD′ (24.1 kcal/mol). The Markovnikov product 4′ results in the *6-endo-tet* annulation P1′ as the final product, which agrees with the experimental observation of the *6-endo* product well, indicating that the difluoroiodotoluene-promoted fluorocyclization of unsaturated alcohol plausibly operates the “fluorination first and cyclization later” mechanism.

### “Cyclization First and Fluorination Later” Mechanism

Alternative to the “fluorination first and cyclization later” mechanism, we have considered the possibility of nucleophilic addition of the oxygen atom of the functional group to the hypervalent iodine activated C=C double bond, followed by fluorination. The calculation results are shown in Figure 3 and Figure 4. The first step is also the activation process by the hypervalent iodine reagent to form the iodine (III)-π unsaturated carboxylic acid intermediate 3. After the activation, with the assistance of another HF molecule as the Brønsted acid, the carboxylate group nucleophilically attacks the C=C double bond to furnish the cyclization, accompanied by the dissociation of the proton, as shown in Figure 3. This cyclization step may also proceed *via* the Markovnikov transition state TSF (−0.4 kcal/mol) to the *5-exo-trig* product 8 (−33.7 kcal/mol) or *via* the *anti*-Markovnikov transition state TSH (18.2 kcal/mol) to the *6-endo-trig* product 10 (−27.3 kcal/mol). In addition to the Markovnikov rule, the high constraint of the [6.3.1] bicyclic structure of the transition state TSH in comparison to the 5,8-fused bicyclic structure of transition state TSF should mainly contribute to the higher free energy of TSH. Specifically, the cyclization transition state TSF (−0.4 kcal/mol) is lower in free energy than in the corresponding alkene fluorination transition state TSB (2.0 kcal/mol, Figure 1), indicating that the carboxylate oxygenation of the alkene is preferred over fluorination in Hara and Yoneda’s system using excess HF. After cyclization, the fluorination process occurs with the assistance of two bridging HF molecules. The fluorination transition state TSG (−42.1 kcal/mol) leads to the *5-exo-trig* product P2, while the transition state TSI (−45.2 kcal/mol) produces the *6-endo* product P1. Although TSG is slightly higher than TSI because a five-membered ring lactone causes more steric hindrance than the five-membered ring lactone, the reaction much preferred the Markovnikov transition state TSF (−0.4 kcal/mol) to the *5-exo* product, which can explain the experimental results well. In sharp contrast to the results of the “fluorination and cyclization later” mechanism, these results strongly suggested that the difluoroiodotoluene-promoted fluorocyclization of unsaturated carboxylic acid should plausibly undergo the “cyclization first and fluorination later” mechanism to the *5-exo* product. Notably, DFT results predict a lower cyclization transition state TSF (−0.4 kcal/mol, Figure 3) than that of the alkene fluorination transition state TSB (2.0 kcal/mol, Figure 1), further supporting the preference of the “cyclization first and fluorination later” mechanism for unsaturated carboxylic acid substrates.

**SCHEME 2:**
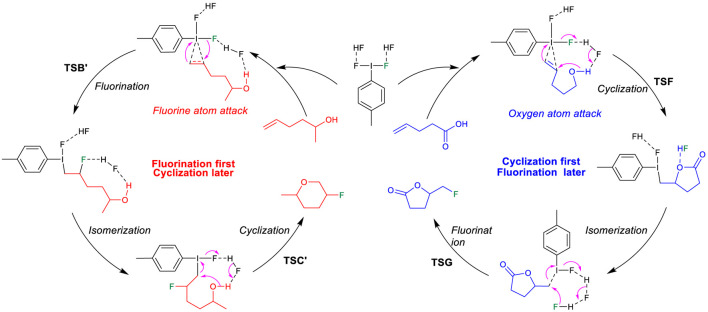
Mechanism-dependent selectivity in difluoroiodotoluene-promoted fluorocyclization of unsaturated alcohols and unsaturated carboxylic acids.

On the other hand, DFT results suggest that after the activation of the unsaturated alcohol (3′ in Figure 4), the hydroxyl-initiated cyclization has to overcome a much higher free energy barrier, either *via* a Markovnikov transition state TSF′ (18.9 kcal/mol) or *via* an *anti-*Markovnikov transition state TSH′ (36.0 kcal/mol). In comparison to the relatively lower transition states (TSB′, 12.0 kcal/mol; TSD′, 24.1 kcal/mol) for the fluorination of the C=C bond in the “cyclization first and fluorination later” mechanism (Figure 2), the unsaturated alcohol is essentially different in the reaction mechanism from the unsaturated carboxylic acid in the difluoroiodotoluene-promoted fluorocyclization and the selectivity of the substrates is actually mechanism-dependent, as summarized in Scheme 2.

### Origin of the Mechanism-Dependent Selectivity

**FIGURE 5:**
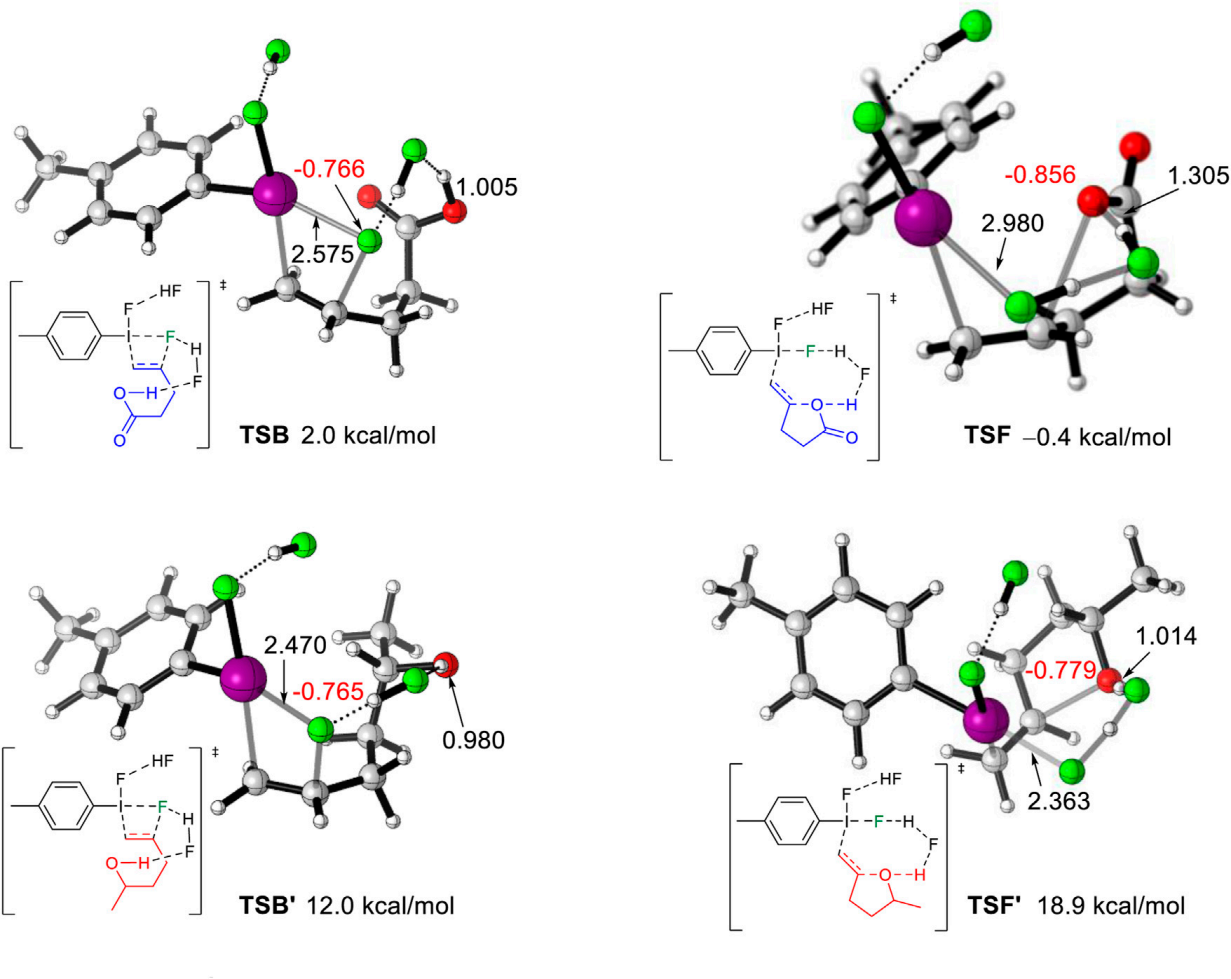
Optimized structures of key transition states.

To compare the mechanism-dependent selectivity in difluoroiodotoluene-promoted fluorocyclization of unsaturated alcohols and carboxylic acids, the key transition states involved in each mechanism are depicted in Figure 5. In the cyclization first mechanism, the cyclization transition state for unsaturated carboxylic acid (TSF, −0.4 kcal/mol) is much lower than that for unsaturated alcohol (TSF, −0.4 kcal/mol). NBO analysis indicates that the nucleophilic oxygen atom of carboxylic acid carries −0.856 NPA negative charge in TSF, while the nucleophilic oxygen atom of alcohol only has an NPA charge of −0.779 in TSF′. The stronger nucleophilic oxygen atom of carboxylic acid can also be well reflected by the degree of the deprotonation in TSF, in which the O…H distance is calculated to be 1.305 Å. In contrast, the alcohol is less deprotonated in TSF**′** with the O-H bond lonely elongated to 1.014 Å. As a consequence, the I-F bond is highly dissociated (2.980 Å) in TSF, and the C=C bond has been highly activated by the hypervalent iodine during the cyclization (C-I bond, 2.489 Å; C=C bond, 1.403 Å). In the case of alcohol, the I-F bond is less dissociated (2.363 Å) in TSF′, and the C=C bond is less activated by the hypervalent iodine during the cyclization (C-I bond, 2.514 Å; C=C bond, 1.410 Å).

On the other hand, in the fluorination first mechanism, the NPA charge of the attacking fluorine atom is lower in TSB (−0.766, carboxylic acid) than in TSB′ (−0.765, alcohol), and the I-F bond is also more dissociated (2.575 Å, carboxylic acid) in TSB than in TSB′ (2.470 Å, alcohol). The difference in TSB and TSB′ can be attributed to the stronger Bronsted acid of carboxylic acid than alcohol in the double acid activation mode (Shu et al., 2019). Due to the more important role of the functional group difference in nucleophilicity for the cyclization first mechanism, the unsaturated carboxylic acid substrates prefer the “fluorination first and cyclization later” mechanism, whereas the unsaturated alcohol substrates would like to operate the “cyclization first and fluorination later” mechanism. The NPA charge and the deprotonation trend of the functional group strongly suggest that the p*K*_a_ property of the substrate should play a crucial role in the preference of the reaction mechanism.

**FIGURE 6:**
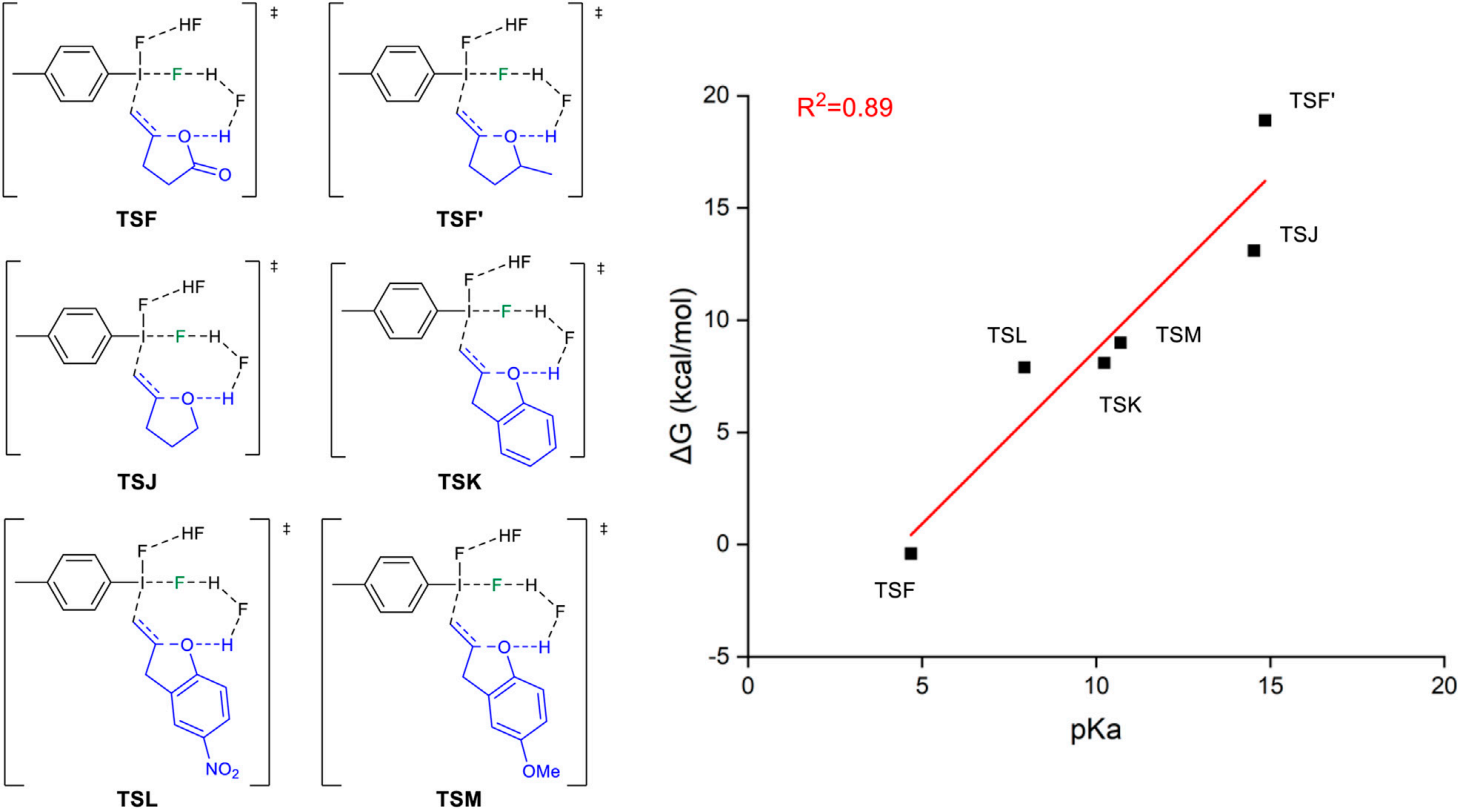
Relationship between *5-exo-tet* cyclized energy barriers of selected reactants with different p*K*_a_ values.

We have designed a series of substrates with different p*K*_a_ values (Yang et al., 2020) to evaluate the free energy barriers of the *5-exo-trig* cyclization. Figure 6 shows the structures of the studied substrates and the method by which the *5-exo-trig* cyclization free energy barriers correlate with the p*K*_a_ value of the reactants. In a general sense, the lower the p*K*_a_ value of the reactants, the easier the deprotonation of the O-H bond. The carboxylic acid has the smallest p*K*_a_ value corresponding to the most facile *5-exo-trig* cyclization. On the other hand, unsaturated alcohols undergo the “fluorination first and cyclization later” mechanism with a larger p*K*_a_ value. If we modified the alcohol to phenol with a lower p*K*_a_ value, the tendency of the *5-exo-trig* cyclization would become more favored. Specifically, phenol with an electronic-withdrawing substituent, such as nitro, will have an even lower free energy barrier for the *5-exo-trig* cyclization. Although the studied reaction is not a stereoselective transformation, it can be expected that the property of the functional group would change the mechanism, highlighting its potential role in influencing the stereoselectivity.

## Conclusion

DFT studies have been performed to understand the unprecedented different selectivity (*6-endo vs. 5-exo*) between unsaturated alcohol and unsaturated carboxylic acid substrates in hypervalent difluoroiodotoluene-promoted fluorocyclization reactions. Both the previously proposed “fluorination first and cyclization later” mechanism and the alternative “cyclization first and fluorination later” mechanism were compared. The unsaturated alcohol prefers the “fluorination first and cyclization later” mechanism, in which the alkene activated by hypervalent iodine will be fluorinated first with a calculated free energy of 12.0 kcal/mol for the transition state (TSB′), followed by the *6-endo-tet* cyclization to furnish the experimentally observed six-membered ring ether product. In contrast, the “cyclization first and fluorination later” mechanism is less plausible for unsaturated alcohol because it has to overcome a higher *5-exo-trig* cyclization transition state (TSF′, 18.9 kcal/mol), leading to the wrong product, five-membered ring ether, after fluorination in the later stage. In contrast, the unsaturated carboxylic acid operated the “cyclization first and fluorination later” mechanism instead of the previously proposed “fluorination first and cyclization later” mechanism. After activation by hypervalent iodine, unsaturated carboxylic acid undergoes a facile *5-exo-trig* cyclization (TSF, −0.4 kcal/mol), followed by subsequent fluorination to produce the experimentally observed five-membered ring lactone product exactly. In contrast, in the “fluorination first and cyclization later” mechanism, the unsaturated carboxylic acid has to overcome a higher fluorination transition state (TSB, 2.0 kcal/mol) and then cyclizes to the wrong product, the *6-endo-tet* lactone. The origin of the mechanism-dependent selectivity was analyzed, revealing that the p*K*_a_ value of the functional group plays a crucial role in the origin of the mechanistic variation. The p*K*_a_ property affects the NPA charge and the deprotonation trend of the functional group, thus influencing the trend of the first cyclization step. The presented mechanistic understanding could be helpful to the design and application of hypervalent iodine reagents in fluorination reactions.

## Data Availability

The Cartesian coordinates of computational structures and figures can be found in online Supplementary Material.
