# Supplementary material for: Mechanism-Dependent Selectivity: Fluorocyclization of Unsaturated Carboxylic Acids or Alcohols by Hypervalent Iodine
Source: Front Chem. 2022 May 10;10:897828. doi: 10.3389/fchem.2022.897828 (PMC9127131; doi:10.3389/fchem.2022.897828)
Supplement: Supplementary file 1 [file DataSheet1.pdf]

Supplementary Material

Mechanism-dependent Selectivity: Fluorocyclization of Unsaturated Carboxylic Acids or Alcohols by Hypervalent Iodine

Jiaqi Su[1,†], Siwei Shu[1,†], Yinwu Li[2], Yong Chen[3], Jinxiang Dong[1], Yan Liu[1,4,*], Yanxiong Fang[1,4,*], and Zhuofeng Ke[2,5,*]

[1]School of Chemical Engineering and Light Industry, Guangdong University of Technology, Guangzhou 510006, China

[2]School of Materials Science and Engineering, PCFM Lab, Sun Yat-sen University, Guangzhou 510275, China.

[3]Guangdong Provincial Key Laboratory of Chemical Measurement and Emergency Test Technology, Institute of Analysis, Guangdong Academy of Sciences (China National Analytical Center, Guangzhou), Guangzhou 510075, China.

[4]Guangdong Provincial Key Laboratory of Plant Resources Biorefinery, Guangzhou, 510006, China.

[5]Guangdong Provincial Key Laboratory of Optical Chemicals, XinHuaYue Group, Maoming, 525000, China

*** Correspondence:**
Yan Liu
yanliu@gdut.edu.cn
Yanxiong Fang
fangyx@gdut.edu.cn
Zhuofeng Ke
kezhf3@mail.sysu.edu.cn

Cartesian coordinates of all the optimized structures

1

C	3.27111600	0.00039700	-0.01057800
C	2.54979300	1.20976200	-0.01075300
C	1.14592600	1.22937800	-0.00508700
C	0.48355200	0.00017800	-0.00083400
C	1.14625600	-1.22907700	-0.00489200
C	2.54990800	-1.20929400	-0.01055200
H	3.08984700	2.15862500	-0.01796600
H	0.58776800	2.16252000	-0.00787300
H	0.58815000	-2.16225200	-0.00753900
H	3.08999700	-2.15816200	-0.01763500
I	-1.68596100	-0.00007900	0.00150900
F	-1.61883100	2.01466600	0.00212300
F	-1.61838200	-2.01481500	0.00187600

C	4.78528600	-0.00015200	0.01632500
H	5.19280800	0.90455400	-0.45756000
H	5.15839800	-0.02718800	1.05381500
H	5.19286400	-0.87973300	-0.50299200

HF

F	0.00000000	0.00000000	0.09267100
H	0.00000000	0.00000000	-0.83404000

Unsaturated carboxylic acid

C	-3.04989300	-0.59084900	-0.39335100
H	-2.64227100	-1.05868100	-1.26506000
H	-4.04468600	-0.82482100	-0.07626100
C	-2.30621800	0.29801400	0.30910000
H	-2.71384100	0.76584600	1.18080900
C	-0.87446100	0.63475800	-0.14727200
H	-0.80672500	0.53677100	-1.21062000
H	-0.63744600	1.63951300	0.13413600
C	0.11868300	-0.33356700	0.52176700
H	-0.11833200	-1.33832200	0.24035900
H	0.05094700	-0.23558000	1.58511600
C	1.55044000	0.00317700	0.06539600
O	2.53627100	-1.02945800	-0.01647300
H	3.16800500	-0.81477200	-0.70670600
O	1.85285900	1.18706400	-0.23547900

2

C	1.79887600	2.44757200	-0.21736900
C	0.75908100	2.76231300	0.67877300
C	-0.45606000	2.05848400	0.66717800
C	-0.59268900	1.03165600	-0.26998800
C	0.38780900	0.70588000	-1.21008600
C	1.58245900	1.43547300	-1.17386100
H	0.90532500	3.55067000	1.41870000
H	-1.25934900	2.30273700	1.35884000
H	0.26572100	-0.10906800	-1.91623300
H	2.37570900	1.16837800	-1.87052300
I	-2.39861800	-0.11703500	-0.20386600
F	-1.21351000	-1.82537800	-0.51284500
C	3.14798000	3.12182400	-0.13941700
H	3.41009000	3.59546900	-1.09821500
H	3.17256500	3.88820400	0.64729300
H	3.91634000	2.36248300	0.07359300
F	0.35960700	-2.31825600	-2.35580400
H	-0.28969600	-2.16022600	-1.65551800
C	0.49514600	-1.59364800	3.81318000
C	0.52063700	-1.35204600	2.49440700

H	1.37551000	-1.41915400	4.43734200
H	-0.40103900	-1.96979400	4.31057300
H	-0.36984600	-1.55072300	1.88913500
C	1.71057200	-0.83246000	1.72921300
H	2.59201500	-0.73670300	2.37832100
H	1.48855300	0.17335000	1.34014100
C	2.03845800	-1.74413100	0.52610700
H	2.43678400	-2.70833300	0.88356500
F	-3.37253100	1.56738800	0.16539300
H	1.11886000	-1.97064700	-0.02489900
C	3.08056000	-1.12545000	-0.40806600
O	3.99852300	-0.42047100	-0.02705900
O	2.93897300	-1.41016200	-1.73273100
H	2.09018000	-1.87530600	-1.90002200

3

C	-4.48279100	1.22858800	-0.34986900
C	-4.33417900	0.18000000	0.58129300
C	-3.11194100	-0.48994000	0.72799200
C	-2.01697200	-0.10387100	-0.06590700
C	-2.13968900	0.94254700	-0.99841900
C	-3.36953600	1.60074400	-1.13135100
H	-5.18682100	-0.11535500	1.19542900
H	-3.01389700	-1.30039800	1.45182500
H	-1.28851500	1.24165100	-1.61165800
H	-3.46876100	2.41390500	-1.85271400
I	-0.13991300	-1.13851000	0.10871800
F	2.05417600	-1.82486900	0.69359100
C	-5.81578800	1.92159800	-0.52831900
H	-6.41645100	1.87705200	0.39105400
H	-5.68284500	2.97541500	-0.81211900
H	-6.39760800	1.43545000	-1.32896000
F	3.02329400	-1.25374500	-1.35353800
H	2.66550500	-1.54438100	-0.42840600
C	0.22155800	0.28222400	2.37460900
C	0.80391000	1.19776700	1.53335400
H	0.83463200	-0.46894600	2.87673800
H	-0.82534000	0.37380600	2.66722000
H	0.16721300	1.96778900	1.08355500
C	2.26181100	1.23248300	1.20311200
H	2.71859900	0.26451500	1.45240500
H	2.73344200	2.01172800	1.83026800
C	2.54165500	1.58655900	-0.27787500
H	2.14756200	0.79570000	-0.92690200
F	-0.67570100	-2.44686500	-1.28237600
H	2.07054300	2.54785500	-0.52729300
C	4.06514700	1.75131100	-0.45346500
O	4.63029500	2.78302200	-0.13116300

O	4.73148800	0.67867700	-0.90983300
H	4.10879800	-0.07907400	-1.10455600

4

C	4.44718500	-1.75611000	0.25764000
C	4.41009600	-0.43732100	0.75326900
C	3.28916200	0.38901200	0.58225500
C	2.18847200	-0.14897400	-0.09264100
C	2.17788900	-1.44867000	-0.60965200
C	3.31793000	-2.24742800	-0.42294000
H	5.27446300	-0.04051800	1.28921500
H	3.27340200	1.40778300	0.96730100
H	1.31803500	-1.83898300	-1.15251100
H	3.32862900	-3.26227000	-0.82505800
I	0.43900700	1.08380600	-0.34199800
F	-2.43640800	-0.29066700	-1.33634600
C	5.68545200	-2.61026100	0.43049800
H	6.11046900	-2.49328300	1.43849400
H	5.46467500	-3.67450200	0.26654400
H	6.46622100	-2.31437500	-0.28933800
F	-5.87173600	1.53667400	0.16578000
H	-5.12623500	1.30615300	0.73800300
C	-0.83053000	-0.62339400	0.38978600
C	-1.62081200	-1.27806200	-0.73426100
H	-1.50710800	-0.18047600	1.13092800
H	-0.14934000	-1.33452800	0.87383200
H	-0.94568100	-1.65197700	-1.52324700
C	-2.53156300	-2.42961600	-0.27729300
H	-1.89972400	-3.29532500	-0.02603600
H	-3.16265100	-2.71098200	-1.13216500
C	-3.42814300	-2.10576000	0.94533500
H	-4.09985900	-2.95888500	1.11703100
F	1.95200600	2.57633500	-0.96604700
F	2.58011300	3.35067300	1.25505100
H	2.36285300	3.15924900	0.32204300
C	-4.26967800	-0.86171300	0.73788500
O	-4.03874400	0.20924600	1.31870900
O	-5.26880800	-1.04082900	-0.13054300
H	-5.72488800	-0.16385200	-0.24299600
H	-2.81872700	-1.94962700	1.84329700

5

C	4.13718700	0.31495300	-0.52221400
C	3.83772000	-0.49435700	0.59232600
C	2.55330500	-1.02399000	0.77955400
C	1.55239500	-0.74395200	-0.16578600
C	1.82041300	0.07049400	-1.28182100

C	3.11185000	0.59288100	-1.44765500
H	4.61807500	-0.70639600	1.32581700
H	2.33567300	-1.63719300	1.65459100
H	1.02399700	0.32534900	-1.98100700
H	3.32344200	1.23269200	-2.30669500
C	5.53508600	0.85727800	-0.73050400
H	6.13496700	0.16197200	-1.34117700
H	5.51375100	1.82243800	-1.25688900
H	6.05825600	0.99170800	0.22710300
I	-0.44240900	-1.53161700	0.07649900
F	-2.68638400	-1.97243400	0.50122100
C	-0.26467100	1.43188100	1.25645200
H	0.34928200	1.46171800	0.34514700
C	-1.15550600	2.69625300	1.29892900
H	-2.04082600	2.52514900	1.92885400
H	-0.56064100	3.49480500	1.76403300
C	-1.58578400	3.14395000	-0.12874600
H	-0.72614900	3.59598600	-0.64060700
F	0.61383600	1.41620200	2.35264800
F	-3.67154800	-0.85292900	-1.30940000
H	-3.29236700	-1.37547500	-0.50528900
C	-1.13130600	0.18020600	1.32460100
H	-2.14123800	0.29477300	0.92925300
H	-1.15918100	-0.22596500	2.34052300
H	-2.39133600	3.88595700	-0.04362300
C	-2.04144100	1.95209000	-0.97146000
O	-1.28807200	1.36243600	-1.74080100
O	-3.29334800	1.57520600	-0.67909200
H	-3.46530900	0.64340100	-1.05764400

6

C	-4.31110200	-0.39222900	0.47633500
C	-3.71731400	-1.51053100	-0.14145500
C	-2.38149800	-1.47676500	-0.56890800
C	-1.64128800	-0.30471500	-0.35462300
C	-2.19236500	0.83143000	0.25397300
C	-3.53164900	0.76622000	0.66576500
H	-4.30697700	-2.41536300	-0.30022400
H	-1.93739700	-2.34430200	-1.05837200
H	-1.60767000	1.73896600	0.39664800
H	-3.97692800	1.63964000	1.14541700
I	0.41716300	-0.23587800	-0.93479800
F	3.19900500	-2.41524600	0.19268000
C	-5.76494800	-0.42137900	0.89679800
H	-6.40609000	-0.00991200	0.09969000
H	-6.10346900	-1.44762900	1.09867800
H	-5.93026600	0.18713500	1.79750800
F	3.12185400	0.00796700	-1.17895500

H	3.14234900	0.82943300	-0.58772800
C	1.87345800	-2.80155800	0.28732400
C	0.93169000	-1.68633400	0.73112200
H	1.79678700	-3.58411700	1.06749600
H	1.55310900	-3.23203900	-0.67538100
H	-0.06048700	-2.14157100	0.86013300
C	1.32177800	-0.90918200	1.99679900
H	1.56407900	-1.68873000	2.74094800
H	0.44115100	-0.36534400	2.36475600
C	2.50358100	0.08577100	1.89638000
H	2.83934400	0.30615500	2.92120800
F	-0.44659800	2.16088100	-1.90722500
F	-0.14624900	3.27856300	0.19047300
H	-0.27434800	2.67416600	-1.06363700
C	2.10234000	1.42892500	1.25855600
O	2.77802100	1.89624700	0.30156900
O	1.04757600	1.98233300	1.74774400
H	0.50596000	2.72573300	0.94273900
H	3.33823300	-0.34670600	1.33875400

7

C	-4.14101800	0.51250900	-0.21102200
C	-3.18194900	1.51226200	-0.46491800
C	-1.82939300	1.19270300	-0.66122300
C	-1.43524600	-0.15674900	-0.60279000
C	-2.36893500	-1.17320600	-0.33375300
C	-3.71363800	-0.82982100	-0.14141100
H	-3.49420400	2.55782200	-0.50154300
H	-1.09040100	1.97872900	-0.81548600
H	-2.04592900	-2.20943300	-0.24641600
H	-4.44259900	-1.61408800	0.07254200
C	-5.60198000	0.86653600	-0.03302300
H	-6.13585000	0.80922200	-0.99634200
H	-6.09939400	0.17235900	0.65991600
H	-5.72086000	1.89030100	0.34968000
I	0.63751800	-0.65457200	-0.91905900
F	2.92868800	-1.17492100	-0.84712000
C	0.31268800	0.30228500	2.09698000
H	0.18720300	-0.09552900	3.11779500
C	0.95083300	1.72183000	2.19488700
H	0.16920800	2.43971000	2.47343300
H	1.72495100	1.71146000	2.97463500
O	2.85142300	1.70750400	0.80024200
H	3.17274500	1.66084200	-0.15987000
F	3.64632700	0.97077600	-1.48507700
H	3.41569100	0.00058000	-1.22832100
C	1.12302100	-0.73318100	1.32199000

H	2.18768700	-0.49877500	1.22022900
H	-0.70473000	0.36747100	1.68811100
C	1.59131100	2.16849700	0.87790700
O	1.01029500	2.79580900	0.00241200
C	0.94076000	-2.17325900	1.75502200
H	1.49390700	-2.85647800	1.09437900
H	1.33821400	-2.25938200	2.78387200
F	-0.41019000	-2.53490500	1.77779900

8

C	-4.32136400	1.53387300	0.38277200
C	-3.85776700	0.49370600	1.21255800
C	-2.68585800	-0.22044700	0.92215500
C	-1.97265300	0.14141700	-0.22695100
C	-2.39836900	1.16118000	-1.08646700
C	-3.57576200	1.85605600	-0.76652600
H	-4.42144500	0.23131100	2.10989500
H	-2.34372700	-1.02875100	1.56751900
H	-1.84115100	1.41371900	-1.98791600
H	-3.92070500	2.65214100	-1.42919600
I	-0.16275300	-0.91812100	-0.68246200
F	2.77588300	-1.78713500	1.04367600
C	-5.60866900	2.26333200	0.70470700
H	-5.63345100	3.25813100	0.23736100
H	-6.47961500	1.69837100	0.33311300
H	-5.73569800	2.38356400	1.79052200
F	2.67101300	-1.89583200	-1.47829800
H	2.78810400	-1.99213100	-0.53378900
C	0.83722400	1.09990600	-0.94974700
C	2.32879600	1.09289400	-0.67599700
H	0.31656700	1.78680700	-0.26996900
H	0.66957900	1.41205800	-1.98943500
H	2.84355300	0.32227300	-1.26481300
C	2.99989900	2.47490800	-0.84112800
H	2.29394900	3.26526500	-0.54545000
H	3.30449700	2.64694300	-1.88095400
C	4.17255200	2.40581500	0.14908800
H	5.06501000	1.94758500	-0.30694000
F	-1.43304600	-2.67981500	-0.31354400
F	-1.24375300	-2.71063700	2.12311100
H	-1.34182300	-2.82766100	1.16080900
C	3.68281900	1.45757700	1.23855300
O	4.10649700	1.25039400	2.34533400
O	2.57135100	0.75762900	0.74549200
H	2.63086900	-0.83889200	1.11314700
H	4.47265600	3.36497200	0.58738400

9

C	-4.14743900	-0.26924000	0.47079300
C	-3.43470200	-1.11661500	1.34244000
C	-2.11317000	-1.49562400	1.06354400
C	-1.49288600	-1.02021700	-0.10561700
C	-2.18169700	-0.17044100	-0.98894600
C	-3.50138700	0.19839500	-0.69111400
H	-3.91806600	-1.48082500	2.25117900
H	-1.57410700	-2.14777200	1.75218600
H	-1.68871100	0.22603800	-1.87487800
H	-4.03543600	0.86611600	-1.36979200
I	0.55044000	-1.59670000	-0.52698100
C	-5.58306500	0.11129600	0.76350500
H	-5.82314100	1.10368500	0.35565800
H	-6.27759900	-0.61126500	0.30358500
H	-5.78218100	0.11774300	1.84481900
F	4.15828900	1.12627800	0.74845100
H	4.10479200	0.19305300	0.99860600
C	1.42975000	0.22701100	0.41079400
C	0.48212200	1.38010500	0.56038600
H	2.25928600	0.43190000	-0.26707100
H	1.81500900	-0.14822500	1.36137800
H	-0.45027600	1.09145700	1.06993100
C	1.19196200	2.55161000	1.30422800
H	2.27448000	2.50095300	1.12801800
H	1.01097200	2.49060800	2.38465500
C	0.55824100	3.77953000	0.63272500
H	-0.34318800	4.12312600	1.16497700
F	2.85637600	-1.88704800	-0.74621500
F	3.80021400	-1.27762800	1.28473800
H	3.41525900	-1.60531200	0.35853200
C	0.11903800	3.27821000	-0.74688800
O	-0.21439600	3.91300700	-1.71811100
O	0.12848800	1.89424600	-0.74836900
H	1.23233700	4.63726600	0.52217900

10

C	4.00228000	-0.89428200	-0.27012900
C	3.77509700	0.33928300	0.37411900
C	2.49255200	0.89780400	0.46631400
C	1.42619900	0.18455100	-0.09861500
C	1.60960400	-1.02210500	-0.78649000
C	2.90699600	-1.55539200	-0.85664600
H	4.61795600	0.88459200	0.80323400
H	2.33179500	1.88357600	0.90274300
H	0.78186900	-1.53866500	-1.26797100
H	3.06561800	-2.49343500	-1.39195200
I	-0.53007100	1.03156200	0.04307200

F	-3.45987900	-0.08424400	-1.80578100
C	5.39361200	-1.48815900	-0.32712700
H	5.46072000	-2.27792800	-1.08840900
H	6.14369800	-0.71709300	-0.55745100
H	5.66962000	-1.93171000	0.64395600
F	-3.60426700	1.65036500	0.03644500
H	-3.63117400	1.09512000	-0.74188000
C	-2.61358400	-1.01527100	1.25549900
C	-1.21993300	-0.52740400	1.62689700
H	-2.99288100	-1.72232600	2.01276700
H	-3.32794900	-0.20058600	1.10743200
H	-1.27906300	0.09691300	2.52763800
C	-0.28278500	-1.72093000	1.83839900
H	-0.38327200	-2.05618600	2.88311400
H	0.76545500	-1.43557500	1.69413000
C	-0.64346500	-2.91274600	0.89510900
H	0.24860400	-3.42699300	0.52269200
F	0.43095600	2.55123800	-1.24775600
F	1.41337300	3.75858500	0.65373600
H	1.05847200	3.37648700	-0.17073200
C	-1.48682500	-2.54047300	-0.31216400
O	-1.32825000	-2.91755400	-1.45119900
O	-2.57850800	-1.72031100	-0.03698200
H	-3.11864800	-0.81151700	-1.27528500
H	-1.24313700	-3.64728300	1.45783100

11

C	-4.14324900	-0.26335500	0.00987900
C	-3.57452300	-1.11194800	0.97943600
C	-2.21498600	-1.45687500	0.93252800
C	-1.41270900	-0.95099400	-0.10730500
C	-1.95933100	-0.10605700	-1.08868500
C	-3.31557200	0.23558300	-1.01654800
H	-4.19856900	-1.49874000	1.78740800
H	-1.78770800	-2.10204400	1.70153000
H	-1.33057200	0.32051700	-1.86823700
H	-3.72961900	0.91785400	-1.75994300
I	0.68413300	-1.45381300	-0.14228000
F	4.17467500	-0.04517100	-0.99659600
C	-5.61147500	0.10017200	0.05796200
H	-6.02514000	-0.03679100	1.06701800
H	-5.76984600	1.14443000	-0.24848500
H	-6.19212900	-0.53580100	-0.63070900
F	4.19031500	1.41983800	0.98511300
H	4.29533100	0.87348100	0.18960700
C	1.66327300	1.50502500	-0.48574500
C	1.36945900	0.58575700	0.68597300
H	2.26297700	2.32745600	-0.06139200

H	2.27128900	1.02703500	-1.25642200
H	2.30544500	0.24624600	1.13353600
C	0.33843500	1.18019300	1.65278900
H	0.78389400	1.22268800	2.65679400
H	-0.56457100	0.55841700	1.72656400
C	-0.09209300	2.60019100	1.19062200
H	-0.95793600	2.95656000	1.75892100
F	3.01773300	-1.73248300	0.06155200
C	-0.45571600	2.61953700	-0.29060200
O	-1.46485100	3.09388700	-0.76628800
O	0.46198500	2.00520400	-1.11393700
H	3.66675500	-0.86857000	-0.53781000
H	0.74067000	3.30357900	1.35180600

P1

C	0.15422100	3.18034600	0.02957800
C	-0.69501500	2.90568300	1.11779900
C	-1.60078400	1.83077200	1.09139600
C	-1.65332300	1.01542500	-0.04950100
C	-0.83810400	1.28184900	-1.16217000
C	0.05802900	2.35856100	-1.11171200
H	-0.64368800	3.53350300	2.00968600
H	-2.23681700	1.62624700	1.95142500
H	-0.88737900	0.65459100	-2.05097400
H	0.71684400	2.54043900	-1.96067400
I	-2.91711900	-0.71053900	-0.07030800
F	2.71321600	-4.12808700	-0.15633700
C	1.15593600	4.31361900	0.06869400
H	1.23285300	4.74400400	1.07749000
H	2.14670000	3.95572900	-0.24874500
H	0.86193900	5.12091900	-0.62146800
F	2.91256500	-1.92902400	1.10801600
H	2.92206100	-3.35502300	0.36510100
C	1.55036800	-1.15436300	-0.73170000
C	1.62762800	-1.30974800	0.77419700
H	1.89099400	-2.06862100	-1.22781400
H	0.49486900	-0.99010500	-0.98869700
H	0.86195300	-2.02672100	1.08758000
C	1.56838100	0.02221900	1.48957300
H	1.66276100	-0.11982000	2.57502500
H	0.57996900	0.45722400	1.28945300
C	2.67612900	0.94448000	0.95433700
H	2.55082300	1.97434000	1.30765800
F	0.15488000	-3.73686300	-0.24713200
C	2.77790500	1.00041000	-0.56783100
O	3.30147900	1.92520200	-1.16019400
O	2.32738500	-0.06656800	-1.29711100

H	1.05288600	-4.03491600	-0.24760600
H	3.65545800	0.58795500	1.31043200

P2

C	-0.45284200	3.10333400	-0.50781800
C	-1.49134500	2.93579900	0.42780600
C	-2.15045600	1.70339100	0.57925900
C	-1.75470900	0.62372500	-0.22362200
C	-0.73101400	0.75718100	-1.17545500
C	-0.09580100	2.00008300	-1.30719800
H	-1.78825200	3.77745100	1.05689800
H	-2.94898200	1.59233700	1.31151000
H	-0.38816800	-0.08886700	-1.76711500
H	0.72573900	2.09502100	-2.01750100
I	-2.70638200	-1.28421400	0.03236100
C	0.30478000	4.40701600	-0.62781200
H	1.36553100	4.24401600	-0.38242900
H	0.26175400	4.79697600	-1.65679700
H	-0.10284000	5.17212900	0.04807800
F	3.39337500	-2.22617900	0.95858000
H	3.83393400	-1.63785100	-0.70016000
C	1.98467300	-2.29463000	0.74338100
C	1.40360300	-0.89535900	0.83943100
H	1.82454500	-2.71490700	-0.25477200
H	1.55431100	-2.94493900	1.51884200
H	0.36692000	-0.93265500	0.47774600
C	1.48705700	-0.22746100	2.22531300
H	2.40663100	-0.55410300	2.73244700
H	0.62028300	-0.48266500	2.84836300
C	1.57493500	1.25927700	1.85193800
H	0.57646200	1.69313000	1.68255800
F	1.25934100	-1.73268300	-2.21949000
F	3.82528600	-1.68275300	-1.64171000
H	2.17511600	-1.53804100	-2.08261800
C	2.28841800	1.23706200	0.50282400
O	2.87997600	2.12424600	-0.06943800
O	2.16166200	-0.02287700	-0.04787700
H	2.11633200	1.89339000	2.56363800

TSA

C	-3.84274600	1.69116000	-0.29773900
C	-3.89691200	0.62386200	0.62392500
C	-2.85594800	-0.30904400	0.71718600
C	-1.75188000	-0.16988700	-0.13849300
C	-1.66159300	0.88076600	-1.06395200
C	-2.71457000	1.80402100	-1.13527300
H	-4.76333400	0.52520000	1.28040000
H	-2.90510400	-1.12293100	1.44036900

H	-0.78745600	0.98587700	-1.70577900
H	-2.65591800	2.62704300	-1.84940900
I	-0.15129500	-1.59762100	-0.06263000
F	1.96334600	-1.61712100	0.87284800
C	-4.98269600	2.68042700	-0.39577600
H	-5.43745500	2.86290200	0.58857400
H	-4.64470900	3.63995900	-0.81115600
H	-5.77434500	2.29087300	-1.05704100
F	3.22387300	-1.13591500	-1.07285500
H	2.75883900	-1.35501000	-0.20578200
C	0.16134400	-0.02707400	2.50769100
C	0.53608900	1.04270500	1.75876800
H	0.90034800	-0.74057900	2.86970900
H	-0.87835200	-0.16214000	2.80860900
H	-0.23738700	1.73377600	1.40821300
C	1.94196500	1.36373000	1.35823700
H	2.61309600	0.54920500	1.65657500
H	2.25450600	2.28602400	1.87997600
C	2.07515200	1.63018200	-0.16461900
H	1.86872800	0.70393600	-0.71501300
F	-1.20897400	-2.74221400	-1.31690200
H	1.36310000	2.40997300	-0.46971400
C	3.49379100	2.15052100	-0.46100000
O	3.78908500	3.32290700	-0.30770500
O	4.40335800	1.22719200	-0.82774600
H	3.98832900	0.32803100	-0.91706600

TSB

C	4.45914500	-0.83457400	0.48228100
C	3.62091000	-0.16358500	1.39555200
C	2.31670200	0.21698800	1.05038000
C	1.85973100	-0.07641500	-0.24554200
C	2.67121800	-0.73592900	-1.18471200
C	3.96591200	-1.11590800	-0.80908400
H	3.99314400	0.06604100	2.39554200
H	1.69065300	0.77083600	1.74689800
H	2.30346400	-0.94474700	-2.19022900
H	4.60605400	-1.62939400	-1.52895100
I	-0.11014200	0.51735800	-0.79195500
F	-2.60218700	0.28096500	-1.39775000
C	5.87241900	-1.21402300	0.86704300
H	5.94651900	-1.43671300	1.94107000
H	6.22066900	-2.08946200	0.30043300
H	6.56594100	-0.38347600	0.65404200
F	-2.96758000	2.03320000	0.11814000
H	-2.88946400	1.28473900	-0.58234500
C	-0.67965500	-1.91970800	-0.55904900

C -1.98626800 -1.95339900 -1.06170400
H -0.51993900 -2.04609400 0.51270900
H 0.12001400 -2.24181000 -1.23025400
H -2.11083600 -1.95719400 -2.14563600
C -3.23489600 -2.21954800 -0.28899500
H -3.55129400 -3.23434300 -0.60508700
H -4.00454500 -1.52757600 -0.65814200
C -3.14631700 -2.15337600 1.25127400
H -4.12417500 -2.44127800 1.66132300
F 0.60021300 2.46215000 -0.92040800
F 0.91569400 2.89272000 1.61777300
H 0.80308500 2.87811700 0.67744700
C -2.80355800 -0.72284000 1.65990400
O -1.68418300 -0.37923000 2.01573600
O -3.85647300 0.08873600 1.48762500
H -3.51529000 1.00503400 1.25607600
H -2.37854700 -2.83613700 1.63796000

TSC
C 4.31701100 1.11465100 -0.44738600
C 3.73665000 0.92288700 0.82218400
C 2.60636100 0.11144900 0.99505900
C 2.04507500 -0.51225600 -0.13164800
C 2.59088500 -0.33134600 -1.41404500
C 3.72572700 0.48007500 -1.55763400
H 4.17326600 1.41664500 1.69224400
H 2.15577200 -0.00637400 1.97733600
H 2.14222900 -0.80791600 -2.28526000
H 4.15405500 0.62518900 -2.55099800
C 5.55940600 1.96332400 -0.61114500
H 6.46710600 1.34780100 -0.49623600
H 5.59841100 2.42587400 -1.60769800
H 5.60216100 2.75840700 0.14690200
I 0.30304000 -1.73294800 0.09238500
F -2.27289700 -2.64844900 0.30107200
C -0.71060700 1.31940100 0.87170100
H 0.08579200 1.61434100 0.16772900
C -1.68882100 2.49720700 1.10315200
H -2.37938100 2.18888600 1.89849600
H -1.07084700 3.32774600 1.47784200
C -2.48329400 2.92814600 -0.13904800
H -1.81460300 3.17017000 -0.98103700
F -0.09792200 1.04014900 2.11012800
F -3.66787900 -1.21794600 -0.97074800
H -2.90131100 -2.06774800 -0.25417000
C -1.40818200 0.08479700 0.38836700
H -1.70847400 0.00668700 -0.64896600
H -1.95912500 -0.52157900 1.09934000

H	-3.02677500	3.85817400	0.08728600
C	-3.51416700	1.89977500	-0.65528700
O	-4.11391000	2.06965800	-1.70764400
O	-3.64563900	0.83983800	0.14739300
H	-3.81100100	-0.19847400	-0.44688200

TSD

C	4.25862300	0.71617700	0.28825700
C	3.81642800	0.62010900	-1.04822100
C	2.53306600	0.14843400	-1.35296300
C	1.68108200	-0.22122800	-0.29880200
C	2.08816400	-0.13828800	1.04504900
C	3.38184100	0.32516700	1.32066600
H	4.48822400	0.91158600	-1.85762000
H	2.20503800	0.06914700	-2.39018300
H	1.42690700	-0.47300800	1.84336300
H	3.71357800	0.38706400	2.35867300
I	-0.30063400	-0.89560700	-0.69623000
F	-2.75684100	-0.68138100	-1.29091400
C	5.65658000	1.19829000	0.60775500
H	6.33754200	0.34178100	0.74395700
H	6.06199200	1.82027800	-0.20267400
H	5.67269200	1.78016800	1.54068600
F	-3.21972100	-1.39015100	0.92265300
H	-3.14367000	-1.06027500	-0.03203400
C	-1.96461500	1.33152700	-1.81991400
C	-0.71496500	1.61843000	-1.26622300
H	-2.89533700	1.60894500	-1.33253800
H	-2.03179900	0.95793200	-2.83998900
H	0.13388500	1.59636200	-1.95731900
C	-0.48541200	2.43234600	0.00392300
H	0.06827600	3.33821600	-0.29471300
H	0.18336000	1.87937000	0.67938800
C	-1.75108000	2.85743500	0.78181700
H	-1.42474300	3.36617800	1.70179100
F	0.31364900	-2.79153700	-0.09925800
F	0.25990700	-2.26009400	2.44091800
H	0.28908700	-2.61744100	1.56360700
C	-2.66180800	1.68493200	1.15788100
O	-3.85088700	1.64837700	0.87670000
O	-1.98196600	0.70188900	1.77499700
H	-2.53204400	-0.13720700	1.68591500
H	-2.35694700	3.56446400	0.20032900

TSE

C	-4.37039800	1.00774500	-0.14478700
C	-3.90784300	0.44157800	-1.34885100

C	-2.72873800	-0.31735800	-1.39381500
C	-2.01341500	-0.51437000	-0.20277300
C	-2.43695600	0.04120700	1.01520700
C	-3.61482700	0.80235400	1.02694100
H	-4.47163100	0.60107200	-2.26965900
H	-2.37838100	-0.73659300	-2.33622100
H	-1.85007400	-0.09250200	1.92159200
H	-3.94915300	1.24550700	1.96673600
C	-5.65922400	1.80009500	-0.10538800
H	-6.51215200	1.14104000	0.12703300
H	-5.62415800	2.57924900	0.66934600
H	-5.86413800	2.27725800	-1.07418000
I	-0.20098300	-1.64029700	-0.24036700
F	2.74160100	-2.30654500	0.15841800
C	0.69971600	1.57263100	-0.00292600
H	-0.18740300	1.75240600	0.61865600
C	1.71771000	2.76345400	0.16555300
H	1.29310300	3.66029200	-0.30365900
H	1.86902500	2.94610500	1.23952600
O	3.63065100	1.37682500	0.19599200
H	3.97128800	0.41614500	-0.44730600
F	3.99864100	-0.60890600	-0.98362400
H	3.34266200	-1.65085300	-0.30215500
C	1.39082800	0.30894200	0.40689900
H	2.07940600	-0.14223600	-0.29285600
H	0.39862800	1.51112900	-1.05686500
C	3.05424800	2.35926700	-0.49658800
O	3.44990600	2.84643500	-1.54640700
C	1.62588000	0.01305200	1.86264300
H	1.94186600	-1.02860700	1.99633400
H	2.43240600	0.68885700	2.18685400
F	0.47914300	0.28320900	2.62597500

TSF

C	-4.37039800	1.00774500	-0.14478700
C	-3.90784300	0.44157800	-1.34885100
C	-2.72873800	-0.31735800	-1.39381500
C	-2.01341500	-0.51437000	-0.20277300
C	-2.43695600	0.04120700	1.01520700
C	-3.61482700	0.80235400	1.02694100
H	-4.47163100	0.60107200	-2.26965900
H	-2.37838100	-0.73659300	-2.33622100
H	-1.85007400	-0.09250200	1.92159200
H	-3.94915300	1.24550700	1.96673600
C	-5.65922400	1.80009500	-0.10538800
H	-6.51215200	1.14104000	0.12703300
H	-5.62415800	2.57924900	0.66934600
H	-5.86413800	2.27725800	-1.07418000

I	-0.20098300	-1.64029700	-0.24036700
F	2.74160100	-2.30654500	0.15841800
C	0.69971600	1.57263100	-0.00292600
H	-0.18740300	1.75240600	0.61865600
C	1.71771000	2.76345400	0.16555300
H	1.29310300	3.66029200	-0.30365900
H	1.86902500	2.94610500	1.23952600
O	3.63065100	1.37682500	0.19599200
H	3.97128800	0.41614500	-0.44730600
F	3.99864100	-0.60890600	-0.98362400
H	3.34266200	-1.65085300	-0.30215500
C	1.39082800	0.30894200	0.40689900
H	2.07940600	-0.14223600	-0.29285600
H	0.39862800	1.51112900	-1.05686500
C	3.05424800	2.35926700	-0.49658800
O	3.44990600	2.84643500	-1.54640700
C	1.62588000	0.01305200	1.86264300
H	1.94186600	-1.02860700	1.99633400
H	2.43240600	0.68885700	2.18685400
F	0.47914300	0.28320900	2.62597500

TSG

C	-4.02868900	-0.47639100	0.52020300
C	-3.31199500	-1.32687800	1.38546300
C	-1.98829300	-1.69943700	1.10872800
C	-1.37618700	-1.21696000	-0.06149100
C	-2.06427600	-0.36394400	-0.94215100
C	-3.38361900	0.00072300	-0.63805600
H	-3.79239800	-1.69955000	2.29217300
H	-1.44551500	-2.35071600	1.79351000
H	-1.57440800	0.03522400	-1.82734300
H	-3.91643200	0.67200600	-1.31392500
I	0.66634000	-1.70387700	-0.46375900
C	-5.46547200	-0.10282600	0.81557800
H	-5.71063600	0.88906900	0.40963900
H	-6.15716200	-0.82777900	0.35520100
H	-5.66337500	-0.09931400	1.89713000
F	3.54352000	1.67043800	0.93627100
H	3.99837500	0.65817900	1.07575100
C	1.72593100	0.53883200	0.37162400
C	0.58427400	1.48613000	0.45459000
H	2.36081600	0.50721400	-0.50437300
H	2.06758800	0.04327900	1.27211900
H	-0.25412300	1.00586600	0.99073700
C	0.88896000	2.85904700	1.12256200
H	1.88698300	3.19202900	0.82375500
H	0.85088200	2.78430900	2.21564700

C	-0.21631800	3.72008800	0.49468300
H	-1.16234200	3.65142900	1.05660500
F	3.39509100	-1.57257300	-0.70297400
F	4.27980800	-0.48510500	1.19501800
H	3.82198400	-1.13610600	0.11804100
C	-0.44284400	3.08088500	-0.88015400
O	-1.01496600	3.52086000	-1.84780200
O	0.12019500	1.81526200	-0.87993700
H	0.03729800	4.78090900	0.38215500

TSH

C	4.09197000	-0.82193900	-0.18282100
C	3.79759500	0.37173200	0.51079100
C	2.49179000	0.87052300	0.58623400
C	1.47051300	0.14870200	-0.05919200
C	1.72417400	-1.02873000	-0.78616000
C	3.04238500	-1.50293400	-0.83216600
H	4.60516700	0.92647700	0.99176500
H	2.28000300	1.82364600	1.06826100
H	0.91867100	-1.56558800	-1.29020400
H	3.25788900	-2.41488000	-1.39158200
I	-0.49211700	0.92767800	-0.00533900
F	-3.51199700	0.30903300	-1.55667600
C	5.50289200	-1.36551300	-0.21399200
H	5.66398900	-2.00757900	-1.09128300
H	6.24231400	-0.55199700	-0.23116000
H	5.70329800	-1.97269000	0.68472800
F	-3.18814000	1.74163300	0.34899200
H	-3.42779000	1.20410600	-0.44919700
C	-2.52238700	-0.85953800	1.82216900
C	-1.12947400	-0.92275300	1.86687500
H	-3.12864200	-1.76049900	1.82578500
H	-3.04330500	0.09634100	1.88862200
H	-0.61063400	-0.16926800	2.46923100
C	-0.42323800	-2.23240900	1.58779100
H	-0.31072400	-2.79576100	2.53105600
H	0.59546500	-2.02262100	1.23594700
C	-1.17347000	-3.12069200	0.54697700
H	-0.48415600	-3.88523000	0.16753200
F	0.19373700	2.46893200	-1.26295900
F	1.28073300	3.77171700	0.66436500
H	0.91843300	3.42242400	-0.14392500
C	-1.72089500	-2.30706900	-0.64412600
O	-1.14737400	-2.27133800	-1.73732600
O	-2.82325900	-1.63676500	-0.36771400
H	-3.19662100	-0.59999600	-1.14006100
H	-2.01621900	-3.62709000	1.04002400

TSI

C	-4.14879000	0.15242100	0.31068600
C	-3.65737000	-0.82915100	1.19349800
C	-2.38061300	-1.38709000	1.02696400
C	-1.59372000	-0.96138400	-0.05712600
C	-2.05394600	0.01819900	-0.95491400
C	-3.32591700	0.56985700	-0.75507600
H	-4.27568600	-1.15700300	2.03106300
H	-2.00902100	-2.13285600	1.72905700
H	-1.42766100	0.37266700	-1.77103600
H	-3.67121300	1.34950400	-1.43517400
I	0.39503000	-1.69584200	-0.27626300
F	4.50161700	0.19917400	0.25820000
C	-5.53208400	0.73921000	0.48993500
H	-5.89757900	0.59776700	1.51669100
H	-5.53869000	1.81438500	0.25880900
H	-6.25027300	0.25345300	-0.19117200
F	3.37612000	1.68255800	1.62383200
H	3.97346500	1.01005200	1.04112300
C	1.98567600	1.08534800	-0.74216000
C	1.59409400	0.51633200	0.59595400
H	2.72329200	1.87127600	-0.52342100
H	2.45561800	0.34012900	-1.38589400
H	2.26870800	-0.17078600	1.08536100
C	0.57795800	1.29651800	1.37573500
H	0.94165600	1.38311100	2.40738000
H	-0.39472400	0.77941700	1.40307300
C	0.40056300	2.69034800	0.72013000
H	-0.39562000	3.26811200	1.20094800
F	3.24286200	-1.68187300	-0.32615500
C	0.05181800	2.53142500	-0.75525500
O	-0.87562200	3.06332800	-1.32756700
O	0.84706300	1.63884100	-1.44504700
H	3.84253900	-0.87515000	-0.06254100
H	1.35564900	3.22726100	0.82545200

Unsaturated alcohol

C	-3.28232700	0.40380100	-0.08136100
C	-2.22981200	-0.42727200	-0.05170400
H	-3.16889600	1.44692200	-0.38885300
H	-4.28542800	0.07218200	0.19314900
H	-2.37850300	-1.46567000	0.26788300
C	-0.81498500	-0.04485300	-0.39993800
H	-0.78783700	0.99764400	-0.75225100
H	-0.43555200	-0.67588400	-1.22190500
C	0.14662500	-0.22331400	0.79759200
H	-0.10738400	0.49423700	1.59495600

H	0.00253000	-1.23108400	1.22924500
C	1.63845900	-0.06702200	0.42762700
O	2.03541800	-1.02598900	-0.56787300
H	1.83457200	-1.90212100	-0.20482000
C	1.97872400	1.31311200	-0.13941000
H	1.43964000	1.48454400	-1.08163200
H	1.69757500	2.09786700	0.57829500
H	3.05651500	1.38340600	-0.34139300
H	2.22931800	-0.22084200	1.35348000

2’

C	1.48916100	2.79381900	0.22380600
C	0.68928500	2.43723300	1.32694900
C	-0.41091600	1.58235600	1.18237300
C	-0.70725100	1.10595200	-0.10088000
C	0.04633300	1.45094600	-1.23108400
C	1.14771100	2.29678100	-1.04934800
H	0.94226800	2.81382300	2.31928000
H	-0.99422100	1.27432800	2.04627600
H	-0.16907000	1.02717600	-2.20793000
H	1.76039500	2.55947900	-1.91254800
I	-2.29639200	-0.27779700	-0.32287400
F	-0.81463700	-1.75565400	-0.48041100
C	2.72384900	3.64775500	0.40574700
H	2.67318700	4.23596700	1.33256400
H	3.62066300	3.00860300	0.46054200
H	2.86269300	4.33476800	-0.44149500
F	0.75836900	-1.24753100	-2.31943300
H	0.15605300	-1.48458200	-1.59962500
C	-0.66181300	-1.63232300	2.70026500
C	0.64962500	-1.34762100	2.65034000
H	-1.10421800	-2.33339100	1.99061500
H	-1.32121200	-1.18763300	3.44955100
H	1.06533600	-0.62982300	3.36830500
C	1.62503400	-1.92515200	1.65810100
H	1.13949200	-2.69336000	1.04269500
H	2.45264800	-2.41553500	2.20288300
C	2.22333400	-0.85162200	0.72398800
H	1.42202300	-0.44900700	0.09338700
F	-3.60805200	1.20389600	-0.15648700
H	2.62630200	-0.01095200	1.31422800
C	3.33446100	-1.40019200	-0.19466500
O	2.89450000	-2.53418800	-0.94513700
H	2.20119400	-2.19997400	-1.54499600
C	3.86429200	-0.29431400	-1.12618300
H	4.67081400	-0.68399600	-1.76384100
H	3.04306400	0.05401500	-1.77039800
H	4.24680800	0.56516800	-0.55131900

H	4.15879800	-1.77642000	0.43581200

3’

C	-4.46990600	1.27675200	-0.19415400
C	-4.36855600	0.03726200	0.47079500
C	-3.15444800	-0.66150100	0.51747700
C	-2.01964700	-0.11389000	-0.10704600
C	-2.09623100	1.12292000	-0.77125300
C	-3.31838100	1.80910600	-0.80864900
H	-5.25164100	-0.38398700	0.95534600
H	-3.09324200	-1.61999100	1.03565400
H	-1.21239800	1.54664000	-1.25045300
H	-3.38144700	2.77049500	-1.32204900
I	-0.14205800	-1.18345900	-0.08435900
F	1.98036100	-1.95528300	0.49081100
C	-5.79448400	2.00484800	-0.26868200
H	-6.39642000	1.83180400	0.63512700
H	-5.64970400	3.08739800	-0.39296200
H	-6.38321900	1.64768000	-1.12994000
F	3.19828300	-0.92785100	-1.26800300
H	2.73868300	-1.40514300	-0.50823300
C	0.09802600	-0.31391400	2.44440800
C	0.62709700	0.80197400	1.84203700
H	0.75603700	-1.12171000	2.76981200
H	-0.95457500	-0.35555100	2.72904300
H	-0.05993600	1.60799300	1.55977200
C	2.08341000	1.00077700	1.56707200
H	2.60857300	0.03816400	1.60926900
H	2.48750100	1.63071100	2.38333700
C	2.37388100	1.68678000	0.21781800
H	2.03613400	1.02771900	-0.59365200
F	-0.62583100	-2.13884200	-1.76292200
H	1.82490900	2.64143200	0.14057000
C	3.88893100	1.93634300	0.01990700
O	4.65166200	0.76643900	0.29185200
H	4.29522500	0.07762600	-0.31307700
C	4.16116200	2.46623300	-1.39872500
H	5.23497500	2.66230700	-1.52732000
H	3.85486200	1.70447900	-2.13213900
H	3.60194100	3.39492200	-1.59984000
H	4.21905600	2.68445700	0.76293300

4’

C	4.66736800	-0.48424200	0.85197200
C	3.95369200	0.72345400	0.98518500
C	2.66400700	0.88403600	0.45652900
C	2.09737800	-0.21011600	-0.20612200

C	2.76729200	-1.42763700	-0.37252500
C	4.05641800	-1.55300200	0.16925500
H	4.41108600	1.56198700	1.51357300
H	2.12585600	1.82605600	0.55281400
H	2.31357600	-2.25983000	-0.90917000
H	4.59486700	-2.49475700	0.04777300
I	0.11097700	-0.00138500	-0.99616200
F	-2.61237600	-1.61023000	-0.83592800
C	6.07169500	-0.61556200	1.40233900
H	6.18321900	-0.05590100	2.34235200
H	6.33295100	-1.66718200	1.58742800
H	6.80774500	-0.20934100	0.68903900
F	-5.12665400	-1.42556300	-0.23923700
C	-0.43083700	-1.88625500	0.14636500
C	-1.92826900	-2.03815800	0.38947900
H	0.11055200	-1.81272000	1.09757700
H	-0.03701300	-2.72569400	-0.43873200
H	-2.17524400	-3.10949400	0.46080100
C	-2.52030000	-1.31585100	1.59994800
H	-2.12488000	-1.86054300	2.47381800
H	-3.60582400	-1.48676000	1.59177800
C	-2.23996000	0.18940700	1.75882600
H	-2.56237300	0.48663100	2.76845300
F	0.94867400	1.83837200	-1.88553100
F	0.69125300	3.26186200	0.07139800
H	0.78662600	2.78875300	-0.78118500
C	-3.00658800	1.10209200	0.76968400
O	-4.41241100	1.03786400	1.01668200
H	-4.75359400	0.22265800	0.61012400
H	-1.15953100	0.39114700	1.70140200
H	-2.79021200	0.77676200	-0.26692800
C	-2.56738900	2.55851400	0.92878900
H	-3.15936400	3.19766300	0.25899300
H	-2.74946900	2.88629000	1.96421800
H	-1.50094000	2.68605300	0.69726200
H	-4.24566000	-1.51135000	-0.57290400

5’

C	-4.31903600	1.31789700	0.36959500
C	-3.80399600	1.03680800	-0.91242900
C	-2.70181600	0.18865600	-1.08306600
C	-2.10004200	-0.39275200	0.04635200
C	-2.59558200	-0.12755800	1.33469400
C	-3.70227200	0.72249000	1.48642100
H	-4.27189600	1.48981500	-1.78878400
H	-2.30469200	-0.00100300	-2.07867600
H	-2.12892600	-0.57776200	2.21212700
H	-4.09094000	0.92612500	2.48620500

C	-5.49022000	2.26202600	0.54007000
H	-6.00767200	2.08897900	1.49437700
H	-5.14973100	3.31088900	0.53085800
H	-6.21681600	2.14725300	-0.27770000
I	-0.40696000	-1.73231200	-0.16293300
F	1.65381400	-2.79230200	-0.31241400
C	0.57164100	1.29508600	-0.48604500
H	-0.15071800	1.48096500	0.32609000
C	1.75021700	2.28686700	-0.39816500
H	2.37701800	2.12571800	-1.28618300
H	1.31201200	3.29233300	-0.47362900
C	2.59983200	2.13392800	0.89333100
H	2.07488100	1.49507600	1.62331100
F	-0.10111600	1.50374300	-1.70494700
F	2.96104100	-1.68634500	1.30344000
H	2.43897500	-2.23415100	0.60402100
C	1.10480200	-0.10874400	-0.43049300
H	1.71677600	-0.30122500	0.44718800
H	1.62127900	-0.42815800	-1.33712400
H	2.72152900	3.11353300	1.38072300
C	4.01842100	1.54416400	0.63178300
O	3.98589800	0.37310200	-0.18198600
H	3.74638200	-0.39576900	0.38264400
H	4.46271400	1.31380100	1.61862700
C	4.91611200	2.56143800	-0.08330800
H	5.01217400	3.48244200	0.51139300
H	5.91333600	2.13013100	-0.24503300
H	4.49400400	2.82103500	-1.06625000

6’

C	-4.23173000	-0.07680400	0.67528200
C	-3.65293500	-1.35132000	0.53117600
C	-2.35097600	-1.50332200	0.02703500
C	-1.63194600	-0.35398300	-0.31779200
C	-2.17228600	0.93233000	-0.20244600
C	-3.47483800	1.05161600	0.30035800
H	-4.22620300	-2.23878500	0.80559700
H	-1.92309200	-2.49857200	-0.09415200
H	-1.60137300	1.80750300	-0.49679600
H	-3.90813700	2.04798800	0.40445100
I	0.37314300	-0.53790800	-1.03760700
F	3.33648200	-1.47642600	0.77339100
C	-5.64603700	0.08349100	1.19140900
H	-5.98435900	-0.81793800	1.72170900
H	-5.72259100	0.94119800	1.87604300
H	-6.34572600	0.26616900	0.35918600
F	3.36645900	-0.41194000	-1.58688500

H	3.51095400	-0.73686300	-0.71452200
C	2.20383600	-2.35066400	0.84537600
C	0.88412600	-1.60505000	0.91699400
H	2.34693900	-2.93263600	1.77043600
H	2.26239300	-3.01918700	-0.02659700
H	0.11355700	-2.39004600	0.95201100
C	0.69373000	-0.67295000	2.12821300
H	0.63728500	-1.33337000	3.01317700
H	-0.28713200	-0.18911200	2.02917600
C	1.75049300	0.41993900	2.41085800
H	1.43563600	0.92757600	3.33740000
F	-0.43040600	0.85053800	-2.65834200
F	0.12222900	2.85588500	-1.50055000
H	-0.10211700	2.04123200	-2.05532800
C	1.90127600	1.49713300	1.32119900
O	0.59782000	2.00820300	1.05335800
H	0.58252700	2.40020600	0.15220800
H	2.73463100	-0.02782000	2.60509700
C	2.85435300	2.61497900	1.77351000
H	3.85699400	2.21464600	1.99545600
H	2.95022800	3.37288500	0.98219900
H	2.45711900	3.10219300	2.67690700
H	2.33805200	1.03138300	0.41710700

7’

C	-4.32838200	0.89721000	-0.18705800
C	-3.77897700	0.42322800	-1.39384600
C	-2.56142700	-0.27537100	-1.41249900
C	-1.88880400	-0.51350300	-0.20304400
C	-2.41285900	-0.04675200	1.01501900
C	-3.62471400	0.65849300	1.01107100
H	-4.30400000	0.61167200	-2.33249800
H	-2.14286100	-0.61854100	-2.35966500
H	-1.86358400	-0.20123000	1.94337000
H	-4.02950900	1.03227100	1.95378000
C	-5.65355300	1.62946700	-0.17265600
H	-5.68118200	2.38734400	0.62374400
H	-5.84599200	2.12554700	-1.13476400
H	-6.48470000	0.92838000	0.01125900
I	-0.00863000	-1.58022000	-0.20394600
F	2.22765500	-2.35341300	0.02405000
C	0.53574600	1.54442300	0.18604900
H	-0.34287600	1.70980000	0.82258300
C	1.55536600	2.70554500	0.38926800
H	1.05643500	3.63198400	0.05997500
H	1.77375800	2.81802700	1.46143300
O	3.77051800	1.63601100	0.28979100
H	3.84367300	0.82527100	-0.25656500

F	3.50103800	-0.77639700	-1.15146200
H	3.00743900	-1.53614600	-0.64457700
C	1.20407000	0.23207700	0.51680000
H	2.05266000	0.02181400	-0.12608900
H	0.20097400	1.53670100	-0.86026000
C	2.90448100	2.57309000	-0.36537900
C	1.57044800	-0.02767000	1.95640900
H	1.96318500	-1.04729700	2.06594900
H	2.35431300	0.70449800	2.20958800
F	0.47060900	0.16176200	2.80954800
C	2.72522800	2.25865600	-1.85866900
H	2.04285800	2.98275900	-2.32963300
H	3.69720300	2.30574700	-2.36947100
H	2.32913300	1.24350600	-2.00014900
H	3.39286800	3.56052300	-0.27521600

8’

C	-4.23093500	0.40905300	-0.18855900
C	-3.42712300	0.66287200	0.94043200
C	-2.11642100	0.17309100	1.04090200
C	-1.61429800	-0.57355300	-0.03168700
C	-2.37958600	-0.85855800	-1.16862800
C	-3.68874900	-0.35742400	-1.23774300
H	-3.83049200	1.25792300	1.76214400
H	-1.49918600	0.38076400	1.91220800
H	-1.97599700	-1.45562100	-1.98626600
H	-4.29654300	-0.57361900	-2.11848300
I	0.41396100	-1.27150200	0.03420200
F	3.74127900	-0.30149000	0.16832000
C	-5.65434700	0.92062900	-0.25704300
H	-5.97160700	1.08406700	-1.29713500
H	-6.35101700	0.19314800	0.19156500
H	-5.76461600	1.86488600	0.29556400
F	3.10483100	-2.31137500	-1.17898700
H	3.47549600	-1.60295900	-0.64910400
C	0.87007500	-0.25801000	-1.91309900
C	1.22023600	1.22517700	-1.73935900
H	0.01838700	-0.37884100	-2.59533500
H	1.72814300	-0.83875100	-2.27138300
H	1.66651800	1.56869200	-2.68832800
C	0.06004100	2.16162400	-1.31331500
H	-0.75868100	1.58479700	-0.87096000
H	-0.33922500	2.71006600	-2.17682600
C	0.68840700	3.08745800	-0.25071300
H	1.19673300	3.94422400	-0.71951700
F	-0.20959700	-1.85827900	2.12486700
F	0.31414400	0.32882300	2.98640200

H	0.13112400	-0.61576700	2.73652500
C	1.72221300	2.18148800	0.41932600
O	2.26101800	1.42087700	-0.72981800
H	3.15246400	0.38307600	-0.25485100
H	-0.05113100	3.46314700	0.46874400
H	1.23645000	1.47535500	1.10962300
C	2.89878100	2.85822400	1.10453800
H	2.54378600	3.39072700	1.99823800
H	3.63133900	2.10263700	1.41968800
H	3.38428400	3.57312800	0.42400200

9’

C	-4.34462300	0.54625000	0.40298400
C	-3.94873700	-0.64236700	1.04656800
C	-2.70770300	-1.23688500	0.77152400
C	-1.84908100	-0.63604000	-0.16491700
C	-2.21997500	0.55204200	-0.81928600
C	-3.46130500	1.13413600	-0.52467900
H	-4.61639700	-1.10511100	1.77613800
H	-2.41408200	-2.15235500	1.28709100
H	-1.53240500	1.03773100	-1.50980300
H	-3.74656600	2.06405300	-1.02090400
I	0.05856300	-1.56426100	-0.59807700
C	-5.69586800	1.16856300	0.68431900
H	-5.65060200	2.26534500	0.61701900
H	-6.44368400	0.82478000	-0.04966500
H	-6.06252100	0.89373000	1.68376300
F	4.14870000	0.11702800	0.98690000
H	3.88481400	-0.80272300	1.15750300
C	1.22873700	-0.13749400	0.66522000
C	0.64310200	1.25269400	0.75612200
H	2.17343400	-0.15971100	0.12339600
H	1.32883000	-0.66292800	1.61888200
H	-0.38745700	1.24121600	1.13614400
C	1.61673000	2.09183300	1.67124100
H	2.48872400	1.49349000	1.96697400
H	1.09936100	2.44376600	2.57320500
C	2.06339900	3.23402600	0.73160100
H	1.40200100	4.10879400	0.82937400
F	2.27219200	-2.34191400	-0.80686300
F	3.24983100	-2.16652900	1.28581400
H	2.82841200	-2.30473200	0.32158700
C	1.87193600	2.61254100	-0.66063000
O	0.62301800	1.88754900	-0.52590700
H	3.10085700	3.54428500	0.91481900
H	2.69386600	1.89633200	-0.84948900
C	1.73817600	3.59195800	-1.81803600
H	0.91721300	4.29724900	-1.62009100

H	2.67306900	4.15840600	-1.93873100
H	1.52989200	3.05903500	-2.75674000

10’

C	-4.21145300	-1.16589000	0.11035400
C	-3.93734100	-0.03000800	-0.67845700
C	-2.66975800	0.56836900	-0.69378400
C	-1.66074700	-0.00243000	0.09498200
C	-1.90486600	-1.10298100	0.92801000
C	-3.18354400	-1.68288100	0.92011800
H	-4.73487500	0.41000000	-1.28033700
H	-2.48949300	1.49821000	-1.23137500
H	-1.12944700	-1.49355900	1.58618500
H	-3.38534600	-2.53894800	1.56671500
I	0.26100200	0.91567900	0.08714200
F	3.48149900	0.74967400	1.37464200
C	-5.58195600	-1.80889800	0.08646000
H	-5.74437500	-2.43770800	0.97336400
H	-6.37550300	-1.04810900	0.04827600
H	-5.69899400	-2.44856200	-0.80424000
F	2.97942500	2.45265500	-0.41525500
H	3.25246200	1.94650100	0.34863200
C	2.58220600	-0.51406500	-1.46329200
C	1.06329500	-0.67567800	-1.37551000
H	2.95422900	-1.04929000	-2.35406600
H	2.89201100	0.53584900	-1.52133800
H	0.62654200	-0.32201600	-2.31858400
C	0.66443800	-2.14609800	-1.08797200
H	0.11397400	-2.55146800	-1.94960200
H	-0.02520200	-2.21337700	-0.23900500
C	1.90537500	-3.02492200	-0.81107400
H	1.60066800	-4.00111500	-0.40648700
F	-0.87022300	2.42425900	1.27188100
F	-1.78081100	3.47816400	-0.75600000
H	-1.46820200	3.14846400	0.10784000
C	2.86406600	-2.36626900	0.18986900
O	3.27460800	-1.03393600	-0.29567200
H	3.43850300	-0.01057200	0.73569100
H	2.44776100	-3.22094800	-1.74803600
C	2.27423400	-2.20699900	1.59332500
H	2.96972300	-1.66234100	2.24514000
H	1.33267800	-1.64367500	1.55808300
H	2.08309000	-3.19855800	2.02958200
H	3.79709500	-2.94851200	0.24484000

11’

C	-4.24948500	-0.09011200	-0.04700200

C	-3.81507300	-1.12738400	0.80068200
C	-2.47795500	-1.55349100	0.79946100
C	-1.56004200	-0.94402100	-0.07315200
C	-1.96783000	0.09586900	-0.92697000
C	-3.30542200	0.51709500	-0.89914300
H	-4.52731800	-1.60027800	1.47982900
H	-2.15950600	-2.34437100	1.48027600
H	-1.24863900	0.60937600	-1.56302000
H	-3.61874700	1.33524600	-1.55078700
I	0.49089500	-1.63539300	-0.06750000
F	3.97184600	-0.47424300	-0.99581500
C	-5.69834600	0.34855800	-0.05675100
H	-6.16906100	0.19528600	0.92508200
H	-5.79218400	1.41003500	-0.32800800
H	-6.27619800	-0.23289300	-0.79443800
F	4.26437700	1.16558000	0.81265300
H	4.26055700	0.51710300	0.08684400
C	1.65889200	1.24426900	-0.84625200
C	1.39732500	0.42996600	0.42784300
H	2.60189800	1.77801100	-0.63423400
H	1.83219000	0.62737500	-1.73225700
H	2.32286900	0.05004800	0.85991500
C	0.50152200	1.17694000	1.40357200
H	0.63277400	0.79117900	2.42286900
H	-0.55747000	1.07485600	1.12692900
C	0.88755700	2.67301700	1.28753000
H	0.36416700	3.25574400	2.05959800
F	2.80547300	-2.12315100	0.11073900
C	0.49378000	3.19528600	-0.12333800
O	0.59275200	2.14250600	-1.12022000
H	3.46439900	-1.28259500	-0.50324400
H	1.96843100	2.78085300	1.45688500
C	-0.94836500	3.69781400	-0.18004400
H	-1.21572300	3.96770900	-1.21152300
H	-1.64019900	2.91527700	0.16540300
H	-1.06411500	4.58506200	0.45961300
H	1.18778400	4.00624600	-0.41326700

P1’

C	-3.94086400	1.19887700	0.13382400
C	-4.14045800	-0.06281500	0.72695500
C	-3.17623700	-1.07802300	0.63128300
C	-1.98885600	-0.82208000	-0.07216500
C	-1.75584800	0.42378900	-0.67496400
C	-2.73749200	1.42136500	-0.56100100
H	-5.06287300	-0.25803600	1.27789000
H	-3.34743900	-2.04495900	1.10254500
H	-0.82269100	0.64034200	-1.19218200

H	-2.55592900	2.39299900	-1.02560000
I	-0.48184400	-2.34499200	-0.19225700
F	5.19297300	-0.04485100	0.09967100
C	-5.00130400	2.27663700	0.22151400
H	-5.57259100	2.19892500	1.15814200
H	-4.55447300	3.28020500	0.16850000
H	-5.71979900	2.19087100	-0.61088900
F	3.36364300	1.23058200	1.27526000
H	4.60105300	0.51230800	0.60898400
C	2.15519000	0.58527600	-0.74188500
C	2.19288600	0.46976600	0.78880400
H	3.18671400	0.74067300	-1.10481200
H	1.78387500	-0.36033800	-1.15321500
H	2.39429700	-0.56426300	1.08345600
C	0.97167300	1.07446300	1.45044600
H	1.06610500	1.00692800	2.54271500
H	0.12262800	0.44324500	1.15536700
C	0.72427900	2.53521900	0.98497900
H	-0.36055400	2.71173900	0.93415700
F	3.55505800	-2.02163000	-0.27862300
C	1.35763300	2.80460300	-0.40263300
O	1.28556900	1.61477200	-1.21701000
H	4.24366700	-1.37757400	-0.19137700
H	1.14648200	3.24821500	1.70835800
C	0.65082100	3.91931700	-1.16616800
H	1.14042200	4.10286900	-2.13249100
H	-0.39695300	3.63674200	-1.34853100
H	0.66351800	4.84723900	-0.57529900
H	2.42411600	3.06300700	-0.26069500

P2’

C	4.04386100	1.30509400	-0.07094400
C	4.28648700	-0.01531400	-0.49702700
C	3.29280600	-1.00345800	-0.42240500
C	2.03049000	-0.66344200	0.08959200
C	1.75679600	0.64444600	0.52270300
C	2.76704700	1.61511200	0.43387500
H	5.26777300	-0.27802100	-0.89826600
H	3.49824500	-2.01779500	-0.76262900
H	0.76864200	0.90091800	0.90289500
H	2.55488100	2.63414800	0.76646300
I	0.47191200	-2.12593600	0.18274500
C	5.13605000	2.35313700	-0.12999700
H	4.71396000	3.36487900	-0.21897200
H	5.75293000	2.33189400	0.78413800
H	5.80851700	2.18076700	-0.98330700
F	-3.60082600	0.60451600	-1.47761600

H	-3.90067600	0.11620200	0.06462300
C	-2.26097400	0.13172100	-1.75391200
C	-1.27506200	1.05257500	-1.05391500
H	-2.21340100	-0.88872500	-1.35737600
H	-2.14346000	0.14855300	-2.84718700
H	-0.26242100	0.66547500	-1.25311400
C	-1.40616000	2.55235000	-1.44834300
H	-2.36050700	2.72518000	-1.96566500
H	-0.58850800	2.87218200	-2.10745400
C	-1.40750800	3.26912100	-0.08276700
H	-0.37780700	3.44863700	0.26502800
F	-2.87948900	-2.51029600	0.07124100
F	-4.02365500	-0.31036300	0.90630400
H	-3.24185400	-1.76466900	0.52830600
C	-2.06803600	2.21965200	0.82289600
O	-1.49810400	0.97209500	0.36730300
H	-1.94813700	4.22591400	-0.09594600
C	-1.80322700	2.34584400	2.31637200
H	-2.24121600	1.48950600	2.84700800
H	-0.72029500	2.36903000	2.51007600
H	-2.25443500	3.27124800	2.70411700
H	-3.15842900	2.20678700	0.63720300

TSA’

C	-4.06066600	1.61817700	-0.40230500
C	-4.11911300	0.52879400	0.49185600
C	-3.03200400	-0.34178200	0.64754000
C	-1.87512000	-0.11856200	-0.11556500
C	-1.78086300	0.95832600	-1.00936700
C	-2.87954400	1.81991000	-1.14441700
H	-5.02604900	0.36221400	1.07625400
H	-3.08726200	-1.17536700	1.34800900
H	-0.86896100	1.12740600	-1.58207400
H	-2.81793700	2.66119400	-1.83706300
I	-0.21570400	-1.48266900	0.03543000
F	1.86426000	-1.50795000	0.99190400
C	-5.25154000	2.53533500	-0.57522700
H	-5.79977500	2.65912500	0.37000200
H	-4.94401000	3.52697000	-0.93581300
H	-5.95691100	2.11708100	-1.31232200
F	3.08466200	-1.47789200	-1.09916600
H	2.68465500	-1.47730800	-0.19783200
C	-0.00652600	0.23634700	2.44780600
C	0.52121200	1.25429600	1.71473700
H	0.64408900	-0.49879600	2.92124100
H	-1.07797300	0.18118500	2.64265600
H	-0.16211900	1.97752800	1.25662600
C	1.97998400	1.44009600	1.44852700

H	2.54445000	0.63215900	1.93142100
H	2.30056700	2.40056700	1.89091600
C	2.31384200	1.45545300	-0.06172200
H	1.90102200	0.55178600	-0.52308700
F	-1.22012000	-2.69335600	-1.21121700
H	1.86616800	2.33070800	-0.56155800
C	3.83803200	1.42711600	-0.32796900
O	4.08609700	1.07722400	-1.68441800
H	3.81051200	0.14078100	-1.73856200
C	4.50227400	2.77775500	-0.05581800
H	5.58466200	2.71361400	-0.23456200
H	4.08222200	3.53913900	-0.73125000
H	4.33393500	3.09575200	0.98445300
H	4.27319700	0.66312700	0.34797100

TSB’

C	-4.34962400	0.67652700	-0.40569800
C	-3.40032100	1.35083600	0.38920300
C	-2.11779100	0.82611400	0.60248400
C	-1.79368900	-0.40528300	0.00866900
C	-2.71757200	-1.10869000	-0.78036000
C	-3.98938300	-0.55636800	-0.98672200
H	-3.66670100	2.30445300	0.84895600
H	-1.40065300	1.34144000	1.24016800
H	-2.45624000	-2.06916500	-1.22629300
H	-4.71561700	-1.09409500	-1.59924100
I	0.16451200	-1.21645100	0.35152500
F	2.48361700	-1.96658700	-0.04687300
C	-5.73703900	1.24775700	-0.60247500
H	-5.71571900	2.34690100	-0.62238800
H	-6.18966100	0.88754100	-1.53726800
H	-6.39996900	0.94660700	0.22570900
F	3.86304200	-0.26996900	0.81962300
C	0.46347600	-1.03707800	-2.10265800
C	1.86152700	-0.88810300	-2.10475000
H	-0.15795900	-0.15305400	-2.25175800
H	0.05350700	-1.98119000	-2.46726200
H	2.46963400	-1.77345900	-2.28178100
C	2.60599400	0.38768500	-2.00284500
H	3.12852100	0.45703700	-2.98032800
H	3.43261600	0.22917500	-1.28799500
C	1.85713500	1.69623100	-1.67646700
H	2.30753900	2.50600600	-2.26966100
F	-0.33107500	-1.21838900	2.38553200
F	0.23767500	1.27275700	2.69098800
H	0.02833600	0.34574200	2.75406700
C	1.97213900	2.12905400	-0.18747800

O	3.33897400	2.31450400	0.15588900
H	3.64213100	1.44789000	0.49757300
H	0.79687300	1.64458500	-1.97297200
H	1.52176300	1.36455900	0.46199000
C	1.21817500	3.44167300	0.04331900
H	1.27907100	3.71674900	1.10416000
H	1.66461300	4.24775500	-0.55968900
H	0.15757200	3.33663500	-0.23550400
H	3.27192400	-1.01868800	0.47280100

TSC’

C	-4.32758900	1.38312600	0.32433100
C	-3.64399200	1.25390100	-0.90098800
C	-2.58001800	0.35352100	-1.05223500
C	-2.18889300	-0.43387300	0.04518500
C	-2.84904600	-0.31998500	1.28124600
C	-3.91590200	0.58255400	1.40731400
H	-3.94863300	1.86523900	-1.75283300
H	-2.04995100	0.28298300	-1.99937400
H	-2.54156000	-0.92724600	2.13278200
H	-4.43493000	0.66618900	2.36424800
C	-5.45621900	2.38063500	0.47879600
H	-6.13129700	2.09599400	1.29841400
H	-5.06234500	3.38545800	0.70602600
H	-6.04495900	2.46168000	-0.44668200
I	-0.52797400	-1.78041800	-0.14404900
F	1.88256500	-2.81549900	-0.28724300
C	0.76090300	1.30818300	-0.52080900
H	-0.07131600	1.41341100	0.19774400
C	1.75350400	2.47467900	-0.31556800
H	2.39373400	2.51803200	-1.20725500
H	1.15382500	3.39467900	-0.27618400
C	2.61290000	2.31035800	0.95734400
H	2.01676200	1.82574600	1.74873500
F	0.21330400	1.40427700	-1.81186900
F	3.25587800	-1.60347100	1.12171900
H	2.55530200	-2.29138500	0.39798900
C	1.39779800	-0.02791400	-0.33463400
H	1.70471700	-0.35167700	0.65291300
H	1.77415900	-0.59075700	-1.18057600
H	2.90037100	3.29961500	1.34283900
C	3.90347500	1.48987300	0.68083900
O	3.63339100	0.42043100	-0.24129100
H	3.63294100	-0.46559200	0.29254500
H	4.25857200	1.05973700	1.63233800
C	5.00276400	2.36739600	0.06897000
H	5.28643000	3.17217500	0.76467200
H	5.88931500	1.75932100	-0.15630300

H 4.65051400 2.82260800 -0.86933300

TSD’

C -3.84264400 1.76899100 -0.21360700
C -3.91032200 0.50916800 0.42056900
C -2.79174200 -0.32918500 0.50484200
C -1.58961800 0.11464200 -0.07370000
C -1.48657700 1.35039900 -0.73708200
C -2.62280900 2.17023900 -0.79324200
H -4.85805700 0.17391100 0.84610500
H -2.86337100 -1.32433700 0.94125300
H -0.54165100 1.65778000 -1.18614800
H -2.56205300 3.13199400 -1.30574000
I 0.11981700 -1.14139500 -0.05439600
F 2.66849700 -1.63295900 0.39120100
C -5.05502500 2.67293500 -0.25629400
H -4.98199600 3.40120500 -1.07617900
H -5.97987700 2.09157000 -0.38315000
H -5.15025400 3.23899400 0.68546400
F 2.97527300 -0.85983000 -1.84217600
H 2.91931900 -1.20699400 -0.89819700
C 2.09682300 -0.44862600 2.14922300
C 0.98811900 0.37152200 1.93825200
H 3.10999500 -0.07500900 2.03724900
H 1.98000300 -1.41840700 2.62879400
H 0.06727300 0.06515900 2.44669400
C 1.06187900 1.84357200 1.58309200
H 0.81221900 2.40064800 2.50417600
H 0.25680500 2.07756800 0.87439600
C 2.39638200 2.37124400 1.02273100
H 2.25603800 3.43527800 0.77350500
F -0.95878000 -2.40501100 -1.34614100
F -2.36055000 -3.44360800 0.52606800
H -1.91373500 -3.15718200 -0.26575100
C 2.91449100 1.62269100 -0.21368500
O 1.84651900 1.48472000 -1.16415900
H 2.10496300 0.67936200 -1.66827900
H 3.17996700 2.32930300 1.79733800
C 4.11629600 2.32864500 -0.85546700
H 4.47412800 1.72893000 -1.70397800
H 3.82278400 3.32518300 -1.21869200
H 4.93984100 2.44061700 -0.13198200
H 3.23373100 0.61585500 0.08501200

TSE’

C -4.34946800 1.03802300 -0.17429500
C -3.96364600 0.31622900 -1.32123300

C	-2.79947200	-0.46593000	-1.33369600
C	-2.01346000	-0.53480900	-0.17157200
C	-2.36667800	0.18010600	0.98562600
C	-3.53164000	0.96235300	0.96955700
H	-4.57790900	0.37129900	-2.22228300
H	-2.51005400	-1.00615000	-2.23493700
H	-1.73337700	0.14441200	1.87077700
H	-3.80766800	1.52383900	1.86432800
C	-5.62358500	1.85621500	-0.16781800
H	-5.57861700	2.65865200	0.58229200
H	-5.81226600	2.30819900	-1.15265400
H	-6.49310400	1.22287000	0.07490800
I	-0.20189800	-1.67133700	-0.16328200
F	2.49402300	-2.37034800	0.15435200
C	0.75861500	1.61935200	0.10752100
H	-0.09830300	1.83020900	0.76099700
C	1.77566300	2.81745200	0.18909700
H	1.31120100	3.70381100	-0.27187300
H	1.96624700	3.04199100	1.24879600
O	3.67198600	1.37419100	0.26442000
H	3.80797700	0.54900800	-0.32942300
F	3.56177000	-0.72979400	-1.09884700
H	3.05322500	-1.67942400	-0.43097300
C	1.46097400	0.37969900	0.52587000
H	2.07286200	-0.14143300	-0.20279400
H	0.40930300	1.51921600	-0.92788900
C	3.11395500	2.45665200	-0.48377700
C	1.67181400	0.04055300	1.96982700
H	1.89273300	-1.03012400	2.06911200
H	2.53674700	0.62967000	2.31424700
F	0.55805600	0.39122400	2.75073900
C	2.98297500	2.13873600	-1.98015400
H	2.52505400	2.98622300	-2.51224900
H	3.97481200	1.94318700	-2.40931600
H	2.38169100	1.23418300	-2.13671600
H	3.78322400	3.33014200	-0.36166300

TSF’

C	-4.29637000	0.64882500	0.25764400
C	-3.28112500	0.93130400	1.19301800
C	-1.98680400	0.40955300	1.04993500
C	-1.70194000	-0.40743400	-0.05713700
C	-2.70229300	-0.72440400	-0.99184500
C	-3.98710600	-0.18478900	-0.83586200
H	-3.50672600	1.56707600	2.05168400
H	-1.22095500	0.61050600	1.79765500
H	-2.48597300	-1.38309700	-1.83517900
H	-4.76334500	-0.42442200	-1.56548300

I	0.28236900	-1.23543200	-0.29614400
F	3.52393900	-0.86298300	0.81186600
C	-5.69619000	1.19370300	0.44520800
H	-6.21699400	1.30083500	-0.51718800
H	-6.29618300	0.51369000	1.07267500
H	-5.67927400	2.17256300	0.94605300
F	2.36049200	-2.33830000	-0.52017300
H	3.06616800	-1.62507900	0.23563200
C	0.40604400	0.05675000	-2.44912500
C	1.10569700	1.24551900	-2.15739200
H	-0.68425000	0.14026600	-2.45472100
H	0.83807500	-0.63775700	-3.17228400
H	2.04549000	1.45498400	-2.66493600
C	0.46755700	2.35114300	-1.37333700
H	-0.41515300	1.96363700	-0.84237900
H	0.10865400	3.10422300	-2.09965100
C	1.44813800	3.03093300	-0.38518600
H	2.19566600	3.61488700	-0.94567700
F	-0.01305000	-2.05530300	1.61516700
F	0.57494800	0.08549600	2.87574400
H	0.39523300	-0.78354500	2.51663000
C	2.18271000	1.95781000	0.42336000
O	2.77379400	1.10858800	-0.57014400
H	3.08531300	0.27121400	-0.09105100
H	0.89987900	3.71968700	0.27322700
H	1.47204700	1.37907800	1.03588000
C	3.27136200	2.52655900	1.34178400
H	2.82271400	3.17672300	2.10816300
H	3.78680500	1.69579600	1.84132200
H	4.00188600	3.10493000	0.75658500

TSG’

C	-4.07500000	-0.52422400	0.54514200
C	-3.42716000	-1.56664500	1.23721700
C	-2.10098900	-1.91987600	0.94796100
C	-1.41282700	-1.22193200	-0.05961500
C	-2.02961000	-0.17196000	-0.76115700
C	-3.35388800	0.16789100	-0.44727800
H	-3.96448000	-2.10803100	2.01833200
H	-1.61363700	-2.72228400	1.50155200
H	-1.47277100	0.39420900	-1.50459300
H	-3.83335800	0.98655600	-0.98796600
I	0.61936500	-1.72251300	-0.50294600
C	-5.51701400	-0.17332400	0.84427400
H	-5.71842400	0.88943300	0.64667200
H	-6.20081400	-0.76221600	0.21040500
H	-5.77163100	-0.38884400	1.89201600

F	3.69761000	1.45077600	0.99136000
H	4.05291900	0.41063100	1.09756600
C	1.78510000	0.48997900	0.38820000
C	0.70968300	1.51064800	0.43898300
H	2.42925800	0.40747600	-0.47781000
H	2.05904800	-0.04581200	1.28886200
H	-0.17175300	1.07255900	0.95168500
C	1.10165700	2.81233400	1.19497500
H	2.12229900	3.09672600	0.92160800
H	1.04475100	2.68478600	2.28318900
C	0.05375900	3.77342600	0.61214700
H	-0.91868700	3.63104200	1.11081400
F	3.32118400	-1.73187200	-0.75889200
F	4.23943200	-0.77263300	1.18143700
H	3.75995800	-1.34629700	0.08990900
C	-0.04129800	3.32945100	-0.86368000
O	0.34736300	1.92474800	-0.88144200
H	0.34242000	4.82900800	0.70806000
C	-1.43193000	3.48014600	-1.47861600
H	-1.44858500	3.08706500	-2.50531600
H	-2.16846900	2.92958700	-0.87444100
H	-1.72110600	4.54207600	-1.50816200
H	0.70087000	3.87564700	-1.47116700

TSH’

C	4.10202300	-1.37045300	-0.18851200
C	3.94650400	-0.19170700	0.57112500
C	2.72100000	0.48376200	0.62833300
C	1.62604600	-0.02959000	-0.09315400
C	1.76531600	-1.18148900	-0.88933300
C	2.99857400	-1.84829600	-0.92214800
H	4.80262300	0.20948600	1.11713100
H	2.62718000	1.42649100	1.16613700
H	0.93271000	-1.54973900	-1.48829800
H	3.11060500	-2.74210000	-1.53894500
I	-0.23477500	1.02260200	-0.01796800
F	-3.34000900	0.91298700	-1.47366500
C	5.42511500	-2.10451300	-0.20923200
H	5.50967800	-2.75208200	-1.09330900
H	6.26945900	-1.39966300	-0.20840600
H	5.53070500	-2.74252300	0.68418400
F	-2.37692200	2.14270200	0.21305800
H	-2.94245300	1.57399600	-0.71474600
C	-2.39921000	-0.36807700	1.95844600
C	-1.11636100	-0.83661000	1.67342100
H	-3.21010500	-1.04189700	2.21426500
H	-2.58700500	0.70116800	2.07902300
H	-0.28754100	-0.33394900	2.18420100

C	-0.90714200	-2.27820100	1.23868800
H	-0.51444000	-2.86199100	2.09038000
H	-0.11099600	-2.30565600	0.47962200
C	-2.18282300	-2.96786100	0.69598600
H	-1.90102100	-3.95993600	0.31187200
F	0.67610100	2.50133500	-1.21500500
F	1.94740300	3.53677000	0.75704800
H	1.52689300	3.27068700	-0.05553500
C	-2.94507800	-2.22840800	-0.42896800
O	-3.53951700	-1.02556600	0.09925600
H	-3.47738900	-0.26129700	-0.57352700
H	-2.88797000	-3.14345000	1.52381600
C	-2.09328400	-1.96769200	-1.67791500
H	-2.67182700	-1.40473800	-2.42107300
H	-1.20680900	-1.37302500	-1.43386700
H	-1.77237900	-2.92447000	-2.11679100
H	-3.78395900	-2.88802000	-0.72121400

TSI’

C	-4.26905500	0.29518600	0.22587400
C	-3.94399600	-0.86299400	0.96048400
C	-2.68846400	-1.47614300	0.84003600
C	-1.74425200	-0.92402300	-0.04199200
C	-2.03481400	0.23586400	-0.78285400
C	-3.29541300	0.83455200	-0.63691300
H	-4.68094200	-1.28949200	1.64370000
H	-2.45212700	-2.36451100	1.42512900
H	-1.28095400	0.67952900	-1.43274100
H	-3.52161300	1.73854000	-1.20573600
I	0.19455600	-1.78788600	-0.24571100
F	4.49634800	-0.32189700	0.06546200
C	-5.63803500	0.93017800	0.34538300
H	-6.05911900	0.78523900	1.35069900
H	-5.59683200	2.00853400	0.13515000
H	-6.34063600	0.47904700	-0.37492000
F	3.66093000	1.16871300	1.63155200
H	4.11705500	0.49244700	0.96339200
C	2.04343400	0.91748200	-0.80054000
C	1.64424300	0.37786900	0.54075100
H	2.99643200	1.44672600	-0.61148800
H	2.26765300	0.12030900	-1.51508100
H	2.24928600	-0.38180800	1.01308400
C	0.71212600	1.24972400	1.31411400
H	0.77750800	1.03586100	2.38740700
H	-0.32490800	1.06780600	0.98493300
C	1.06303800	2.72719900	0.97933600
H	0.40152900	3.39407900	1.55063600

F	2.97380700	-1.99684500	-0.47314700
C	0.89697000	2.99727100	-0.54522500
O	1.03893300	1.77578600	-1.32356700
H	3.68925800	-1.26857100	-0.22884400
H	2.09753100	2.89746300	1.29741300
C	-0.47589800	3.56860500	-0.89476700
H	-0.59769700	3.63196800	-1.98548100
H	-1.27160100	2.92951600	-0.48511500
H	-0.58003300	4.57676700	-0.46761700
H	1.68820100	3.69788500	-0.87023800

J

C	2.55177200	-0.46032100	-0.26146800
C	1.34154900	-0.34399900	0.30641400
H	3.07411200	0.41124800	-0.66684000
H	3.06197600	-1.42286000	-0.33495600
H	0.83130600	-1.22790100	0.69504700
C	0.58266300	0.95786600	0.41988300
H	1.24132100	1.78824900	0.11750200
H	0.30046900	1.13447200	1.47377800
C	-0.71000800	1.01161300	-0.43602000
H	-1.08860600	2.04815100	-0.44923200
C	-1.85345000	0.11006200	0.05524500
O	-1.54695900	-1.28905400	0.05269500
H	-1.20194900	-1.49413900	-0.82986500
H	-0.46438400	0.75319700	-1.48168300
H	-2.09529200	0.35419600	1.10179800
H	-2.75843500	0.31649500	-0.55142600

K

C	-2.29546200	-1.18927700	-0.20309800
C	-2.68674600	0.14006000	-0.41840100
C	-1.76670300	1.18155600	-0.22767300
C	-0.44863100	0.89747400	0.17348800
C	-0.03574500	-0.43770200	0.39752800
C	-0.97808300	-1.46034600	0.20130200
H	-3.00559000	-2.00449000	-0.34477600
H	-3.70567000	0.37374300	-0.73063300
H	-2.06576000	2.22090100	-0.39017000
H	-0.66323300	-2.49183000	0.37574900
C	1.40176900	-0.74841700	0.78501500
H	1.42430000	-1.73588400	1.27644000
H	1.77060900	-0.00265300	1.50442600
C	2.30676400	-0.76370600	-0.43152900
H	2.08208100	-1.53318900	-1.17748900
C	3.30256900	0.10677400	-0.64777200
H	3.52461700	0.89602700	0.07413600
H	3.91738700	0.06268400	-1.54881500

O	0.48246400	1.88918800	0.37506900
H	0.06316300	2.74268900	0.18742500

L

C	1.56081000	0.06052800	-0.00445300
C	1.42964700	1.43216000	0.25445500
C	0.16499800	2.01449800	0.15633600
C	-0.95681600	1.23339000	-0.19155300
C	-0.82306600	-0.15534700	-0.45534900
C	0.44984100	-0.72417500	-0.35521800
H	2.30698000	2.01593400	0.52205200
H	0.03815600	3.08225100	0.34967500
H	0.59177700	-1.78518900	-0.55179500
C	-2.04174600	-1.00379200	-0.78162700
H	-1.70160200	-1.90967000	-1.30974400
H	-2.71639100	-0.45205800	-1.45209100
C	-2.78828700	-1.39380000	0.48008600
H	-2.23328900	-2.02501000	1.18140300
C	-4.03153400	-0.99239500	0.77799100
H	-4.59265100	-0.34669300	0.09862300
H	-4.52301000	-1.29411700	1.70463500
O	-2.20601200	1.77197900	-0.30196000
H	-2.15924000	2.71986700	-0.10168700
N	2.89206400	-0.56843300	0.09079100
O	3.85005300	0.15339400	0.40399600
O	2.97617700	-1.78195700	-0.14711300

M

C	1.74403400	-0.29158800	-0.05286600
C	1.74638900	1.08106500	0.24819100
C	0.54083700	1.80256800	0.18456400
C	-0.66095700	1.17344300	-0.16794700
C	-0.67193700	-0.20992000	-0.47568600
C	0.53280100	-0.91787900	-0.41154600
H	2.66259500	1.59810900	0.52626100
H	0.54214700	2.87139500	0.41499600
H	0.54753800	-1.98306700	-0.64646600
C	-1.97682900	-0.91537300	-0.81213100
H	-1.74436300	-1.82754300	-1.38684600
H	-2.60463600	-0.26723100	-1.44073400
C	-2.73620400	-1.28444500	0.44731800
H	-2.23764000	-2.00447200	1.10462800
C	-3.91961200	-0.76381200	0.80035900
H	-4.41848000	-0.02501000	0.16899600
H	-4.41876500	-1.05157800	1.72771100
O	-1.85939400	1.85140400	-0.24393800
H	-1.69756100	2.77791500	-0.01128000

O	2.85303400	-1.10724900	-0.03188900
C	4.09329900	-0.50162100	0.32398900
H	4.05756500	-0.07832000	1.34412600
H	4.84416800	-1.30033800	0.28275000
H	4.36736700	0.30227700	-0.38299400

TSJ

C	-4.23666300	0.36878200	0.09350100
C	-3.37091100	0.21287100	1.19383800
C	-2.02151000	-0.13084200	1.02312600
C	-1.54500500	-0.32564000	-0.27931300
C	-2.37744000	-0.18785300	-1.39726400
C	-3.72044800	0.16408500	-1.20198700
H	-3.75500700	0.36291400	2.20446300
H	-1.36814200	-0.27075900	1.88323500
H	-1.99978100	-0.35992200	-2.40535500
H	-4.37746900	0.27163100	-2.06712600
I	0.53463200	-0.91854800	-0.53753800
F	3.63272900	-0.92723400	0.59715700
C	-5.69606900	0.71311300	0.29685900
H	-5.84643100	1.27999000	1.22652800
H	-6.08843100	1.30414900	-0.54324800
H	-6.30332300	-0.20467300	0.36693300
F	2.83357100	-0.80128500	-1.55988800
H	3.37127500	-0.90211500	-0.43503900
C	0.68386300	1.10194700	-2.13833300
C	1.55981300	1.81926300	-1.32703400
H	-0.36010300	1.41041200	-2.22288500
H	1.11542500	0.49301700	-2.93447800
H	2.60299100	1.50871600	-1.34256600
C	1.16164800	2.91553400	-0.40277800
H	0.29552800	3.45812700	-0.81319300
H	2.01694300	3.59622700	-0.28916700
C	0.76513300	2.41305100	1.03060700
H	0.42527300	3.29900000	1.58826300
F	0.02547500	-2.45036700	0.85042800
F	0.28404900	-1.09361700	2.95860200
H	0.24240200	-1.72482600	2.23650500
C	1.94641800	1.70636900	1.78183600
O	3.09791700	1.57208100	0.96600800
H	3.33421200	0.60326900	0.89155200
H	-0.10565400	1.74794900	0.94700400
H	2.20417600	2.33102700	2.65743300
H	1.60187400	0.73068500	2.15755500

TSK

C	-0.02340300	3.84432700	-0.07874700
C	0.35100400	2.87730700	0.87563500

C	-0.21901000	1.59662000	0.88464600
C	-1.18646700	1.29913900	-0.08794500
C	-1.59261900	2.23630500	-1.05144900
C	-1.00136800	3.50686100	-1.03788300
H	1.10847300	3.12333700	1.62159200
H	0.06484100	0.85425800	1.62582300
H	-2.34835100	1.98466600	-1.79596700
H	-1.30475300	4.24771300	-1.77984400
I	-1.94939200	-0.68109800	-0.13159500
F	0.52081400	-3.47895000	0.08684300
C	0.59229200	5.22624700	-0.05615400
H	1.64714200	5.18561600	0.25151900
H	0.53037800	5.70981400	-1.04143500
H	0.06432000	5.87133500	0.66555700
F	-1.62711300	-3.26202600	-0.79022800
H	-0.67333700	-3.41244300	-0.37551100
C	-0.67448900	-0.91422600	-2.23638000
C	0.63699800	-1.12574700	-1.78337000
H	-0.90626700	0.04925900	-2.69851000
H	-1.22672300	-1.79203700	-2.57577000
H	0.93526800	-2.15116900	-1.56080500
C	1.66004300	-0.04205900	-1.73533500
H	1.14645100	0.92392500	-1.57636100
H	2.09949300	0.01609500	-2.75096300
C	2.76335800	-0.28769800	-0.72843400
F	-3.16026600	-0.16126800	1.48820700
F	-1.30808700	-0.00858500	3.27223300
H	-2.08123600	-0.04586800	2.72395300
C	2.40291600	-0.93356100	0.48001800
O	1.09775000	-1.25457300	0.66596300
H	0.86839700	-2.31820700	0.50829300
C	3.38275900	-1.17314400	1.46245100
C	4.70820600	-0.77154200	1.23838500
C	5.06845500	-0.12625400	0.04317600
C	4.09242400	0.11056000	-0.93763500
H	3.08708100	-1.66830900	2.38727400
H	5.46385400	-0.96665700	2.00119800
H	6.10068200	0.18113700	-0.12746800
H	4.36532800	0.60097200	-1.87509300

TSL

C	-0.40139300	3.83291900	0.00003000
C	-0.08043400	2.81950600	0.92624700
C	-0.72339700	1.57451600	0.90477700
C	-1.71296400	1.36356300	-0.07047300
C	-2.06837200	2.34857200	-1.00733300
C	-1.40220500	3.57994700	-0.96249900

H	0.69335700	3.00004800	1.67391700
H	-0.47637000	0.79633400	1.62226300
H	-2.84184100	2.16305300	-1.75274800
H	-1.66419200	4.35687400	-1.68256300
I	-2.60434800	-0.55279400	-0.16075100
F	-0.33695000	-3.51491300	0.11299000
C	0.29692100	5.17311700	0.05514500
H	1.35614800	5.05716100	0.32619900
H	0.23362600	5.69936600	-0.90751600
H	-0.16883700	5.81639100	0.81985900
F	-2.43416600	-3.16809100	-0.89766500
H	-1.54425000	-3.38126800	-0.43793200
C	-1.36200500	-0.83628100	-2.34132200
C	-0.11177200	-1.14549600	-1.80809300
H	-1.52376000	0.15390400	-2.77520000
H	-1.98946900	-1.66016300	-2.68395000
H	0.08353600	-2.18306900	-1.53546800
C	0.97629200	-0.13510900	-1.66604400
H	0.51815100	0.85434300	-1.48319800
H	1.45038500	-0.05231000	-2.66375800
C	2.04268500	-0.46761000	-0.64365700
F	-3.80495900	-0.02840100	1.44091400
F	-1.94538200	0.04419800	3.25191700
H	-2.72352300	0.04032500	2.71493400
C	1.65288200	-1.11280000	0.56896400
O	0.36216500	-1.38033300	0.77919800
H	0.03362600	-2.45894100	0.54321700
C	2.64327800	-1.40955900	1.53889400
C	3.97815300	-1.06868600	1.32335700
C	4.33766100	-0.42342100	0.12679100
C	3.38001700	-0.12755000	-0.85631400
H	2.33187900	-1.90352800	2.45865000
H	4.74787500	-1.28963000	2.05966200
H	3.70084900	0.36213700	-1.77432500
N	5.74307000	-0.05179800	-0.10706700
O	6.57005300	-0.32679200	0.77480400
O	6.02188000	0.51971700	-1.17362600

TSM

C	-0.19613200	3.83012400	-0.03955500
C	0.10053300	2.82014700	0.89679600
C	-0.56452000	1.58540200	0.88108500
C	-1.54864600	1.37989200	-0.09790600
C	-1.87813900	2.36284500	-1.04528900
C	-1.19349000	3.58467200	-1.00717400
H	0.87198500	2.99415000	1.64864900
H	-0.33630200	0.81055400	1.60784400
H	-2.64696400	2.18144000	-1.79677900

H	-1.43822200	4.35884700	-1.73680400
I	-2.45574600	-0.53853300	-0.19531200
F	-0.23404400	-3.54945200	0.04529900
C	0.51931300	5.16267900	0.00810700
H	-0.03390400	5.87470400	0.64278800
H	1.52790800	5.05756800	0.43234600
H	0.60239000	5.60951500	-0.99319900
F	-2.32482400	-3.13448100	-0.89750700
H	-1.40213600	-3.37375600	-0.45885200
C	-1.09978400	-0.84533900	-2.22505400
C	0.17602400	-1.17804300	-1.73352600
H	-1.22505400	0.14288400	-2.67652600
H	-1.70096900	-1.66606000	-2.61999100
H	0.38704200	-2.23135000	-1.54436900
C	1.28536400	-0.18651100	-1.63603500
H	0.85346700	0.82222400	-1.50552700
H	1.78492600	-0.17982500	-2.62512700
C	2.29895800	-0.52329800	-0.56268800
F	-3.67991300	0.05771700	1.39403000
F	-1.87109700	0.13071400	3.22131300
H	-2.62736900	0.12716100	2.64777400
C	1.80644300	-1.14893200	0.60109000
O	0.46675100	-1.37900200	0.68902300
H	0.17220700	-2.42078300	0.50243300
C	2.69723400	-1.45261800	1.65021400
C	4.05415000	-1.14081600	1.53241000
C	4.54665800	-0.51336900	0.36815800
C	3.66345800	-0.20527000	-0.68355200
H	2.30950800	-1.92986400	2.54983200
H	4.75558800	-1.37766900	2.33228300
H	4.02136600	0.27109200	-1.59545600
C	6.43252900	0.36724800	-0.80773000
H	7.50653800	0.48823500	-0.62107300
H	6.27993400	-0.26163200	-1.70336100
H	5.97308300	1.35629600	-0.98672600
O	5.89683500	-0.25160500	0.35935800

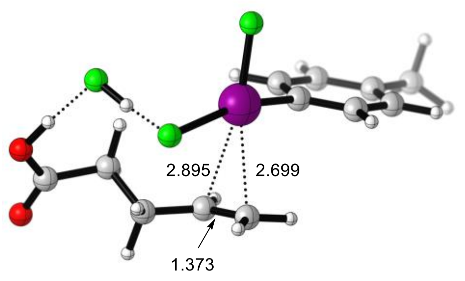

3 -1.4 kcal/mol

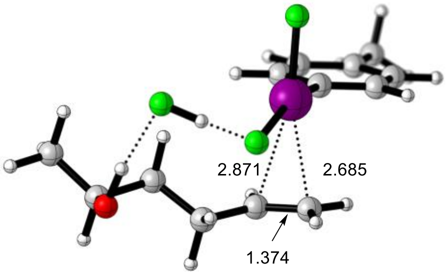

3' 11.6 kcal/mol

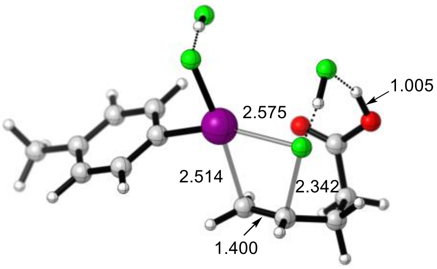

TSB 2.0 kcal/mol

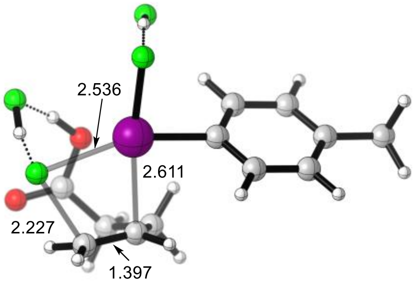

TSD 9.4 kcal/mol

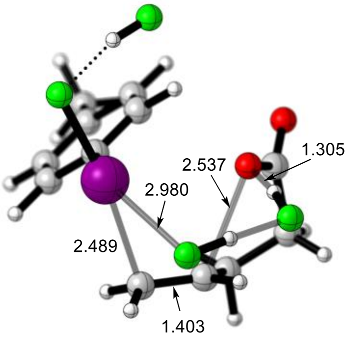

TSF -0.4 kcal/mol

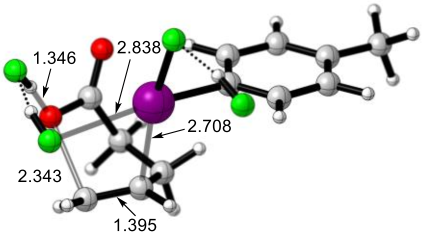

TSH 18.2 kcal/mol

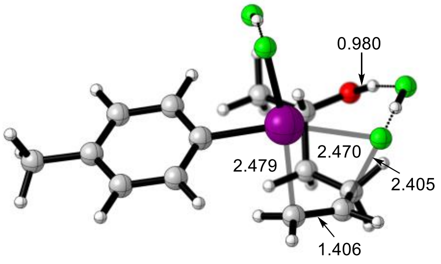

TSB' 12.0 kcal/mol

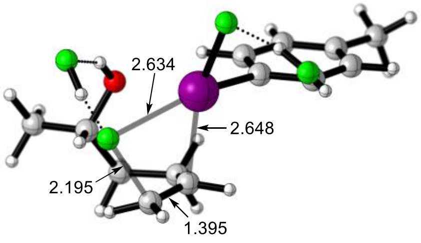

TSD' 24.1 kcal/mol

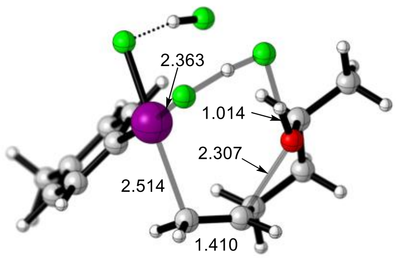

TSF' 18.9 kcal/mol

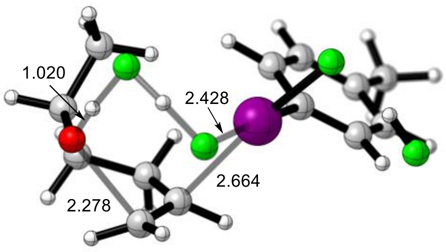

TSH' 36.0 kcal/mol

FIGURE S1. Optimized structures of key intermediates and transition states.
